# A Focus on Regulatory Networks Linking MicroRNAs, Transcription Factors and Target Genes in Neuroblastoma

**DOI:** 10.3390/cancers13215528

**Published:** 2021-11-03

**Authors:** Patrizia Perri, Mirco Ponzoni, Maria Valeria Corrias, Isabella Ceccherini, Simona Candiani, Tiziana Bachetti

**Affiliations:** 1 Laboratory of Experimental Therapies in Oncology, IRCCS Istituto Giannina Gaslini, 16147 Genoa, Italy; mircoponzoni@gaslini.org (M.P.); mariavaleriacorrias@gaslini.org (M.V.C.); 2 Laboratory of Genetics and Genomics of Rare Diseases, IRCCS Istituto Giannina Gaslini, 16147 Genoa, Italy; isabellaceccherini@gaslini.org; 3 Department of Earth, Environment and Life Sciences, University of Genoa, 16132 Genoa, Italy; simona.candiani@unige.it

**Keywords:** neuroblastoma, microRNAs, transcription factors, miR-34, let-7, miR-204, MYCN, PHOX2B, ALK, LIN28B

## Abstract

**Simple Summary:**

Neuroblastoma is a tumor of the sympathetic nervous system that substantially contributes to childhood cancer mortality. Neuroblastoma originates from the neural crest cells that are subjected to genetic alterations during embryonic development. These impairments hit key genes, whose expression is activated/repressed by transcription factors and inhibited by negative regulators, named microRNAs, thereby promoting tumorigenesis. Here, we have focused on the interactions between the transcription factors MYCN and PHOX2B with their target genes ALK and LIN28B and the miRNAs let-7, miR-34 and miR-204, which should act as negative regulators of their expression. In neuroblastoma, the physiologic regulatory networks among these genes and microRNAs are disrupted, leading to a complex and aberrant oncogene expression that underlies the development of the tumor. We also looked into the role of these genetic determinants of neuroblastoma starting from their physiological role in neural crest development and ending with their pathogenic dysregulation that leads to neuroblastoma oncogenesis.

**Abstract:**

Neuroblastoma (NB) is a tumor of the peripheral sympathetic nervous system that substantially contributes to childhood cancer mortality. NB originates from neural crest cells (NCCs) undergoing a defective sympathetic neuronal differentiation and although the starting events leading to the development of NB remain to be fully elucidated, the master role of genetic alterations in key oncogenes has been ascertained: (1) amplification and/or over-expression of *MYCN*, which is strongly associated with tumor progression and invasion; (2) activating mutations, amplification and/or over-expression of *ALK*, which is involved in tumor initiation, angiogenesis and invasion; (3) amplification and/or over-expression of *LIN28B*, promoting proliferation and suppression of neuroblast differentiation; (4) mutations and/or over-expression of *PHOX2B*, which is involved in the regulation of NB differentiation, stemness maintenance, migration and metastasis. Moreover, altered microRNA (miRNA) expression takes part in generating pathogenetic networks, in which the regulatory loops among transcription factors, miRNAs and target genes lead to complex and aberrant oncogene expression that underlies the development of a tumor. In this review, we have focused on the circuitry linking the oncogenic transcription factors MYCN and PHOX2B with their transcriptional targets ALK and LIN28B and the tumor suppressor microRNAs let-7, miR-34 and miR-204, which should act as down-regulators of their expression. We have also looked at the physiologic role of these genetic and epigenetic determinants in NC development, as well as in terminal differentiation, with their pathogenic dysregulation leading to NB oncogenesis.

## 1. Neuroblastoma: Genetic Determinants and Developmental Origin

Neuroblastoma (NB) is a pediatric tumor originating from neural crest (NC)-derived cells subjected to defective differentiation due to genomic and epigenetic impairments. Neural crest cells (NCCs) are a transient population of multipotent cells that migrate from the neural plate border to their final destinations where they undergo differentiation in various types of tissues. Therefore, NB may arise at various sites reached by NCCs during development, mainly in the adrenal medulla (AM) and in the para-spinal ganglia.

NB accounts for about 10% of all pediatric cancers and substantially contributes to childhood cancer mortality, particularly of high-risk patients who are burdened by chemo-resistant relapse, the survival of whom hardly reaches 40–50% [1,2,3,4].

NB is a complex disease showing a remarkable biological and genetic heterogeneity that critically depends on the interaction of several driving and suppressor genes, both coding and non-coding, which act in interrelated pathways to cause or modify the disease-phenotype [5].

In NB tumors, structural and numeric alterations are frequently present at relevant loci such as the deletion of chromosome 1p, 11q, and/or 14q, the 17q gain and several gene alterations, all of which are associated with progression of the disease and poor prognosis [3,6].

It is known that genetic aberrations or dysregulated expression in key oncogenes drives NB tumorigenesis, particularly (1) the amplification and/or over-expression of *MYCN*, strongly associated with tumor progression, invasion and undifferentiated phenotype [2,6,7,8,9,10]; (2) mutations, amplification and/or over-expression of *ALK,* involved in tumor initiation [11,12,13,14], angiogenesis [15] and invasion [16,17]; (3) the amplification and/or over-expression of *LIN28B*, promoting proliferation and suppression of neuroblast differentiation [18,19,20,21,22], all of which correlate with poor prognosis; (4) mutations and/or over-expression of *PHOX2B* involved in regulation of NB differentiation, stemness maintenance, migration and metastasis formation [23,24,25,26,27,28]. Additionally, other mutations and rearrangements affecting other genes, such as *ATRX*, *TERT* and *RAS*, are enriched in high-risk patients [29].

Moreover, the involvement of miRNAs dysregulation in NB tumorigenesis, progression and drug resistance has been demonstrated, as was recently reviewed in [30,31]. This widespread dysregulation of miRNA expression is often caused by both over-expression of the MYCN and by large-scale chromosomal imbalances, which are significantly associated with poor overall patient survival [32].

The role of tumor suppressor (TS) miRNAs is particularly relevant, whose involvement in NB development is well documented (see the paragraph “Tumor suppressor miRNAs in neuroblastoma”). In this review, among these numerous TS miRNAs, we have focused on let-7 [18,20,21,33], miR-34 [34,35,36,37] and miR-204 [38,39,40], which physiologically act as down-regulators of MYCN and PHOX2B and their targets *ALK* and *LIN28B*. 

It is now determined that impaired development and differentiation of NC-derived cells drive NB oncogenesis through genetic and/or epigenetic events occurring in cell-type specific populations with divergent phenotypic states that remodel their regulatory landscapes [41,42].

The latest studies based on single-cell-RNA sequencing (scRNA-seq) with spatial transcriptomics and lineage tracing [43,44,45,46,47,48,49] and transcriptome analyses combined with ChIP-sequencing [41,42,50,51,52] have unraveled the developmental origin of NB and its complex epigenetic landscape.

As most primary NB tumors arise from the AM, investigations on the potential cell population involved in NB development have mainly been focused on the embryonic adrenal development.

These observations have disclosed the specific NC-derived cell populations that may undergo impairments at genetic/epigenetic determinants that control the NB gene expression programs, thereby promoting tumorigenesis.

The first relevant finding revealed that among the NC-derived cell populations, adrenal NB cells transcriptionally resemble immature neuroblasts committed to sympathetic neuronal differentiation or their closest progenitors. Similarly to the adrenal neuroblasts, genetic and epigenetic alterations affecting extra-adrenal neuroblasts committed to the sympathetic chain can be at the basis of NB development in para-spinal ganglia.

A recent study has compared genomic and epigenomic data from primary NBs [29] originating in the adrenal gland vs thoracic sympathetic ganglia, revealing that adrenal NBs are more likely to harbor structural DNA aberrations, including MYCN amplification, whereas thoracic tumors show defects in mitotic checkpoints, resulting in hyperdiploidy [53]. These findings confirm that NB tumors arising from different sites are distinct heterogeneous entities [53].

The comparison of adrenal NBs classified by risk subgroups with normal sympatho-adrenal cells has further highlighted that tumor severity correlates with neuroblast differentiation grade. Specifically, data indicate that high risk tumors derive from early stages of adrenal neuroblast differentiation trajectory, while low-risk tumors arise later during development [45,46,47,48,54], reflecting the differentiation status of the AM development at the time of the onset of genetic or epigenetic hits.

Transcriptome analyses of NBs have identified two cell identities that mark phenotypically divergent states of cellular differentiation: an undifferentiated mesenchymal (MES) identity and a committed adrenergic (ADRN) identity. These cell identities have been found to be the major constituents of either NB cell lines or NB specimens [42,50]. Remarkably, ADRN and MES cell types can spontaneously interconvert into each other by altering their transcriptional states through an epigenetic mechanism of reprogramming [41,42] that confers high plasticity to NB.

Another important aspect emerging from these studies is the discovery of two super-enhancers (SEs) and associated lineage-specific transcription factors (TFs) that form specific core regulatory circuitries (CRC) (see below) and underlie MES and ADRN cell identity states. These CRCs epigenetically define and shape MES or ADRN cell identities, the intra-tumoral heterogeneity and control gene expression programs in NB [42] by endowing enhanced responsiveness to signaling pathways.

The identified ADRN-specific CRC includes important TFs like PHOX2A, PHOX2B, ASCL1, HAND2, GATA2, GATA3, LMO1, TBX2, ISL1 and many others; the tyrosine kinase receptor ALK; and DBH and TH enzymes involved in the metabolism of catecholamines. The MES-specific CRC includes the TFs PRRX1, TWIST1, SNAI2 and MAML3; NOTCH members RUNX1, NFKB and AP-1; TF family members (including JUN and FOS family members), the retinoic acid receptor beta RARB and many others [41,42,50,52,55].

## 2. MicroRNA and Transcription Factor Co-Regulation

MicroRNAs (miRNA) are small non-coding RNA molecules that act as negative regulators of gene expression by a post-transcriptional mechanism based on the recognition of a complementary sequence on the 3′ untranslated region (UTR) of mRNAs. Downregulation of gene expression by miRNAs is mainly carried out through the inhibition of protein translation when the binding to the 3′UTR of the target mRNA occurs with imperfect complementarity, while in the case of perfect match the target mRNA undergoes a cleavage process leading to mRNA full degradation [56,57].

The repressive effect of miRNAs on gene expression is modest, mostly at the level of translation, with little effect on transcript abundance. Nevertheless, miRNAs act in concert with other regulatory processes, such as transcriptional control, to regulate target gene expression at multiple levels and with greater strength [58].

Thanks to the imperfect and dynamic complementarity required, miRNAs act as multi-target gene regulators through their coordinated activities on pathways and networks and have important control functions in fundamental biological processes underlying embryonal development and cell homeostasis such as cell growth, proliferation, apoptosis, differentiation, staminality, reprogramming and cell identity. As these biological processes are commonly altered in many pathologies and during tumorigenesis, miRNAs play a crucial role by exerting their downregulation effects in the context of complex regulatory networks that include TFs and their target genes (TGs).

**Figure 1:**
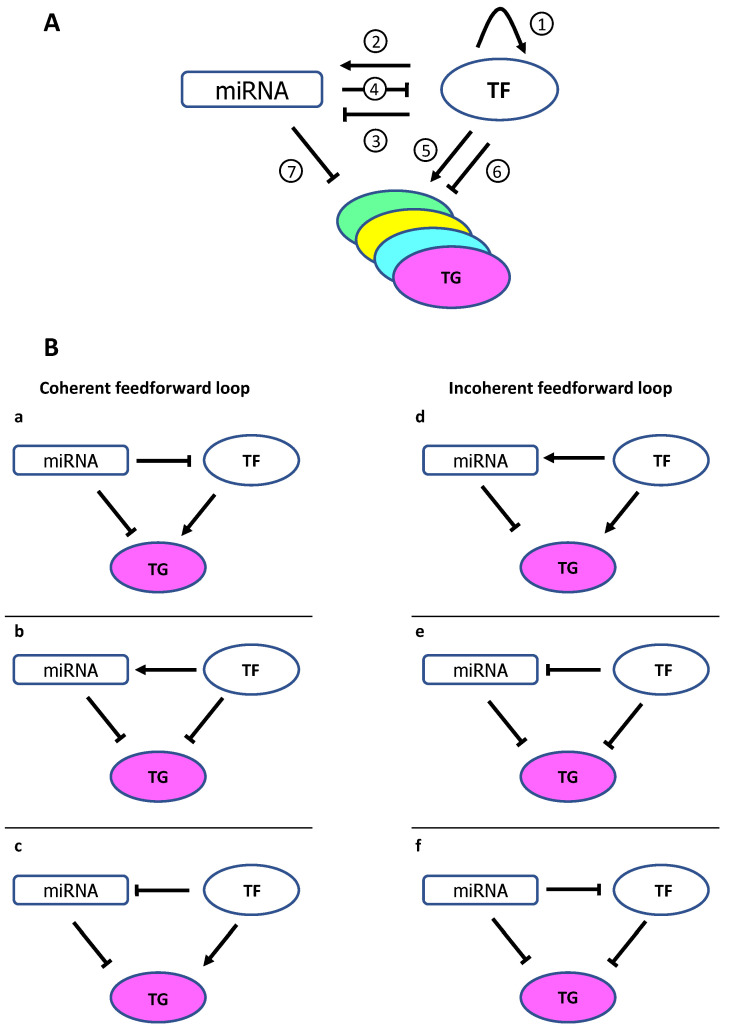
Schematic representation of the most common miRNA-TF-TG auto-regulatory networks. (**A**) Seven main types of regulatory relationships are considered among TFs, miRNAs and TGs. (**1**) TF self-transcriptional activation; (**2**–**4**) Direct reciprocal feedback between miRNAs and TFs; (**5**,**6**) A TF can activate or repress the TG transcription; (**4**,**7**) A miRNA can simultaneously suppress the TF and many of its targets. (**B**) Common miRNA–TF–TG auto-regulatory network motifs. Left side (**a**–**c**): Coherent feedforward loops, in which a TF and miRNA regulate a TG in a complementary direction (either activating or repressing). Right side (**d**–**f**): Incoherent feedforward, in which the TFs and miRNAs have opposing (buffering) effects.

This complex interplay between these two classes of transcriptional and post-transcriptional regulators, TFs and miRNAs, respectively, takes place during development and physiologic cell self-regulation to buffer gene expression and/or to potentiate signaling. Indeed, many miRNA targets in genetic networks are themselves TFs. Furthermore, reciprocal feedback loops have been identified (Figure 1A), including coherent and incoherent feedforward loops, whereby miRNAs and TFs regulate common TGs. In coherent feedforward loops, the most prevalent relationships, TGs are regulated in the same direction (coordinated activation or repression) so that miRNAs and TFs reinforce the activity of each other (Figure 1B, left). In incoherent feedforward loops, miRNAs and TFs carry out opposing functions (buffering effects) (Figure 1B, right), which enables the precise modulation of gene expression to reduce noise and confer stability [59,60].

By physical interaction through 3′UTR mRNA binding, miRNAs can regulate TFs and down-modulate entire functional units to establish and maintain cell phenotype [61]. Many gene sets are reciprocally regulated by strongly interacting pairs of TF-miRNA forming feed forward loops with their common TGs to efficiently suppress functionally related proteins. Moreover, the coherent feedforward loop where the TF activates its TGs and the miRNA simultaneously suppresses this TF and the TGs, is more prevalent (Figure 1B, left), as demonstrated by ChIP-seq experiments that identified this motif enrichment between miRNAs, TFs and TGs [61].

Hence, the influence of miRNAs is mediated not just directly through their primary targets but also indirectly through the action of the TFs that they regulate and of the TF-TG loops. Therefore, the propagation of the signal through TF interactions provides further explanation as to how miRNAs can have a major impact on cell behavior, although they only modestly regulate most of their direct targets [59].

Recent advances in single cell (sc)-transcriptomics have (i) enabled the exploration of cell identity with increasing spatial and temporal resolution, (ii) allowed us to characterize the morphology and transcriptomes for each cell type, and (iii) led to the discovery of super-enhancers (SEs) and their association with lineage-specific TFs to form specific core regulatory circuitries (CRCs) [62,63].

CRCs constitute networks that control cell gene expression programs and confer lineage-specific cell identities. SEs are functional constituent units that drive the expression of TFs, playing prominent roles in both physiology and cancer. TFs belonging to a specific CRC self-regulate and regulate the expression of other CRC TFs under the control of specific SEs in a cross-regulated feed-forward loop.

Structurally, SEs are characterized by the clustering of multiple constituent enhancers in close genomic vicinity to each other that interact with the basal transcription machinery at promoters of the target genes [64].

Physiologically, SEs concentrate multiple developmental signaling pathways at key pluripotency genes in embryonic stem cells and derivatives and endow enhanced responsiveness to the signaling of their associated genes. As SEs are frequently acquired by cancer cells to regulate genes that control cell identity during development and are particularly sensitive to oncogenic perturbation, they provide a program for signaling pathways that promote tumorigenesis [62,63].

There is increasing evidence that miRNAs can also act as key regulators of cell identity, contributing to the determination of cellular diversity, especially in neuronal development [65,66] and a large body of literature documents the crucial role of miRNAs in cancer (see below).

Therefore, it is now clear that a complex and sophisticated regulation of gene expression exists through the interactions between miRNAs, TFs and their TGs in development, physiology and pathology.

## 3. MicroRNAs and Transcription Factors in Neuronal and Neural Crest Development

Most studies that have been conducted on the development of the central nervous system represent a paradigm of a staged approach to move forward with a systematic cell-type classification in the nervous system [66].

Many miRNAs are dynamically regulated during central nervous system (CNS) development and are spatially expressed in adult brains, indicating their essential roles in neural development and function.

The involvement of miRNAs in a time related and spatially diversified regulation of neuronal gene expression is crucial and highly dynamic for neural differentiation and networks. Indeed, miRNAs can regulate cell fate, cell migration, cell polarization and synapse growth during embryonic and early postnatal development. The expression pattern of individual or families of miRNAs in neuronal development show an impressive specificity for distinct developmental stages, regions and cell types [66]. miRNAs are involved in determining the fate of the neuronal progenitor cells (NPCs) by interaction with specifier TFs [67] and operate as master switches of gene expression to sharpen developmental stage transitions by repressing residual transcripts specific to the previous stage [68].

A single miRNA can regulate hundreds of different targets and these targets can also vary according to specific cell types and developmental stages [65], therefore, differential miRNA expression and target regulation may be used to establish and maintain cellular diversity. Once the cells have achieved a mature differentiation state, miRNAs confer robustness to the developmental decision by reducing fluctuations in gene expression and restricting protein levels within a range of values that maintain cell identity [68].

While much is known about NC key genes and TF networks, much less is known about the relationship between miRNAs and genes involved in NC development.

During development, miRNAs facilitate developmental transitions and contribute to progressive changes in gene expression by fine-tuning protein levels, allowing for spatiotemporal protein downregulation, thereby shaping and diversifying the gene expression profiles of different cell types [67]. Indeed, they are efficient molecules to instruct, to determine cell fate decisions and to maintain cellular diversity, including those that affect progenitor cells and cell identity across the developmental trajectories [66].

Particularly in NC development, miRNAs participate in all processes like induction, specification, epithelial-to-mesenchymal transition (EMT), delamination, migration and differentiation through cross talk within the NC gene regulatory network, providing a view of the epigenetic influence on NC development [67].

Recent advances in technology have revealed the genetic and epigenetic determinants controlling the sequential events and the expression programs that lead to differentiated cell types from NC multipotent progenitors within a highly complex gene regulatory network [67] composed of feedback and feed-forward loops, as mentioned above.

At distinct developmental stages, miRNAs are differentially expressed within these networks to downregulate TFs and other key genes to determine different cell fates [67]. For instance, in the phase of NC induction and specification, miR-29b expression is upregulated in neural tube epithelial cells and downregulated in NCCs, thereby promoting neural differentiation and inhibiting NCC lineage; miR-219, miR-218-2, miR-338-3, miR-10b, miR-204a, miR-130b/c, miR-23, miR-24, and miR-196a are upregulated in the NC but not in neural tissue; miR-301a and miR-338 are highly expressed in both tissue types, most likely due to their role in maintaining the stem cell-like phenotype of NCCs [67].

The EMT triggering leads to the activation of a core of TFs, namely SNAIL1/SNAIL2, TWIST1/TWIST2, and ZEB1/ZEB2 that act as E-cadherin repressors and, ultimately, coordinate EMT [69].

Besides other epigenetic control by histone modification and DNA methylation, miRNAs play a key role in the regulation of the EMT process through the downregulation of specifier TF expression [70]. Among multiple miRNAs involved in EMT [69], two core regulatory networks have been defined: the miR-34-SNAI1 (often designed as SNAIL) axis and the miR-200-ZEB1 axis, which employ a double-negative feedback mechanism to repress each other to maintain homeostasis under normal conditions [70].

Many other miRNAs are subsequently involved in the migration and differentiation of NC-derived cells, as reviewed by Weiner [67], among which we find miR-204, let-7 and miR-34, extensively described in dedicated paragraphs below. The most recent investigations by scRNA-seq with spatial transcriptomics and lineage tracing have identified the NC cell progeny involved in normal development and in NB oncogenesis [43,44,45,46,47,48,50].

During the early phases of NC development, different inductive signals (BMP, Notch, FGF and WNT signaling) launch and coordinate the expression of NC specifier genes, principally TFs, in a sequential and tightly regulated manner, activate the EMT machinery and delamination and confer migratory properties to NC cells [71].

From mouse models we have learned that before the NCC migration, controlled by the dorsal aorta, the first segregation signal towards the sympathetic neuronal lineage is represented by the expression of specifier TFs like Foxd3, Sox 9 and Snail2 [72], finely regulated by a plethora of miRNAs, as reported above. NCCs give rise to more specified progenitors that separate different lineages committed to differentiation into numerous different cell types such as the nervous sensory lineage, the melanocyte, osteoclast and chondrocyte lineages [49,73,74], and the last fate split that separates the mesenchymal from the sympatho-adrenal lineage [49], the latter being the most relevant to the origin of NB. During these processes, the gene expression program relies on the sequential and coordinated expression of specific TFs for each lineage that creates diversification circuits and drives differentiation [71].

Among the many TFs involved at different NC differentiation stages, two are actively involved in NB oncogenesis: PHOX2B and MYCN.

In mice, Phox2b is expressed in the precursor of sympathetic neurons, following their aggregation to the dorsal aorta and before expression of all the other sympatho-adrenergic markers [75]. Mycn is initially expressed at high levels, thus promoting ventral migration of NCCs from the neural plate border [76], then it decreases its expression at very low levels in migrating NCCs [77].

The molecular cascade that instructs sympatho-adrenal specification, starts with the activation of BMP signaling [74] that cooperates with Sox10 to activate expression of the sympathetic neuron specifier TFs Ascl1 [78] and Phox2b [75]. Then, Ascl1 and Phox2b activate a hierarchical cascade of transcriptional regulators, which includes Phox2a and Mycn [79], followed by Hand2, Gata2/3 and Trk [80] up to the terminal differentiation with the expression of tyrosine hydroxylase (Th) and dopamine β-hydroxylase (Dbh), all cooperating to mediate cell cycle control, maintenance of survival and differentiation in noradrenergic neuronal subtypes [75,81,82,83,84,85].

Thanks to genetic cell lineage tracing of Ascl1, Sox10, Plp1, Ret and other markers, it has been discovered that sympathetic and adrenergic lineages diverge at an early stage during embryonic development through a split into (1) sympathetic neural progenitor cells (SNPCs) [86] that give rise to differentiated sympathetic neurons, ganglia and the suprarenal sympathetic ganglion (SRG) and (2) Schwann cell precursors (SCPs) [44] that may directly generate glial cells or the specific cell types that colonize the AM, including transient cell populations evolving toward the divergent and terminal differentiated states, chromaffin cells and intra-adrenal neuroblasts [46,47].

During this complex process toward complete differentiation, the spatio-temporal expression pattern of specifier TFs, regulating their own TGs, controls distinct cell populations that can be distinguished by their peculiar protein markers.

## 4. MicroRNAs and Cancer

miRNAs act as genomic switches and are expressed to control cell growth, proliferation, apoptosis, differentiation, stemness and reprogramming [87]. The discovery of thousands of new genes that transcribe miRNAs has broadened our knowledge, demonstrating that altered miRNA expression levels are implicated in various diseases, including cancer.

The causes of the widespread differential expression of miRNA genes in malignant compared with normal cells can be explained by the location of these genes in cancer-associated genomic regions by epigenetic mechanisms and by alterations in the miRNA biogenesis and processing machinery that thereby promote their dysregulated expression [59,88].

Screening of miRNA expression profile in human solid tumor samples and normal controls demonstrated that tumor cells exhibit significantly different miRNA profiles than normal cells of the corresponding tissue of origin [87]. In addition, miRNA expression profile studies have identified specific molecular signatures related to the clinical and biological characteristics of tumors, such as tissue type, degree of differentiation, aggressiveness, and response to therapy, allowing us to define miRNA expression characteristics specifically associated with diagnosis, staging, progression, prognosis and response to treatment [89].

miRNAs are frequently found to be dysregulated in cancer and this contributes to tumorigenesis, cancer cell growth and progression through their ability to act as oncogenes or tumor suppressors (TS), depending on the genomic location of their encoding genes, the presence of their targets and the cellular context. Therefore, miRNAs have aroused interest as potential cancer therapeutics since their altered expression can be inhibited with antagonist oligonucleotides or by replacing TS miRNAs with miRNA mimics [87].

Major examples are the oncogenic polycistronic miR-17–92 cluster located in genomic regions amplified in cancers and the tumor suppressor miR-15a-miR-16-1 cluster located at a region often deleted in cancers. Other examples of TS miRNAs are miR-34 and let-7, which downregulate several pivotal oncogenes and crucial oncogenic pathways involved in multiple stages of the tumorigenic process and in the maintenance of an oncogene addiction and are frequently under-expressed in various types of cancer, including NB [88].

**Figure 2:**
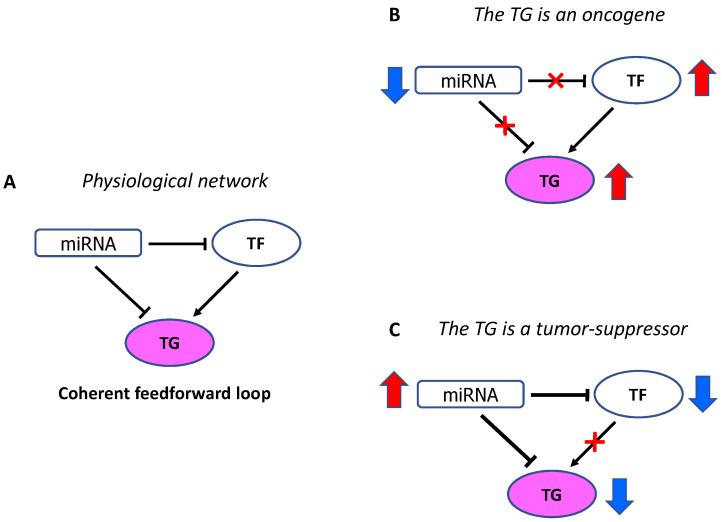
Schematic representation of the most common miRNA-TF-TG feedforward loop disrupted in cancer. One of the most common coherent feedforward loops (see Figure 1(Ba)) frequently disrupted in cancer, especially in NB (**A**) may generate an enhanced oncogene activation when the target gene (TG) is an oncogene (**B**) or a decreased tumor-suppressor function when the TG is a tumor-suppressor (TS) gene (**C**).

From the molecular point of view, in cancer many networks among TGs and the molecules regulating their expression, such as TFs and miRNAs, become aberrant and act in concert to drive oncogenic processes. The consequences of their dysregulation depend on the role of the effective downstream TGs and whether network disruption causes their up- or downregulation with respect to the physiologic balance (Figure 2A). When the TG is an oncogene, the lack of a miRNA leads to an increase in the correspondent oncogenic protein (Figure 2B), while when the TG is a TS an increased expression of a miRNA leads to an inhibition of the TS protein (Figure 2C). In both cases miRNA dysregulation promotes oncogenesis and cancer progression.

## 5. Tumor Suppressor miRNAs in Neuroblastoma

Besides genes having an oncogenic role in NB, the counterpart of regulators acting as TS are not completely known, but an increasing number of TS miRNAs associated with aggressive disease phenotype have been identified as being aberrantly under-expressed in NB, contributing in major ways to the deregulation of the proliferation, differentiation and apoptosis processes. Over the years, many TS miRNAs targeting crucial oncogenes and oncogenic pathways have been disclosed in NB, such as miR-34 [35,36,37,90,91,92], let-7 [18,20,21,93,94,95], miR-96, miR-101, miR-184, miR-204, miR-340, miR-542, miR-591, miR-628, miR-885, miR-20a [34,38,39,96,97,98,99,100,101,102,103] and many more, as recently reviewed in [30,31].

It has been demonstrated that DNA methylation is a common mechanism of miRNA dysregulation in NB and the analyzed miRNAs, including those listed above, are associated with poor patient survival when under-expressed [99]. These findings have led to the identification of a large set of epigenetically silenced miRNA targets that are genes overexpressed in NB tumors from patients with poor survival. Remarkably, this study also revealed a high redundancy, meaning that multiple epigenetically regulated miRNAs often target the same mRNA, providing an additive or even a synergistic impact on reducing target mRNA levels. [99].

The methylation status of a set of miRNAs has also been investigated in a panel of NB cell lines and a subset of hypermethylated and down-regulated miRNAs involved in the regulation of cell cycle, apoptosis and in the control of *MYCN* expression have been identified. Such evidence suggests that *MYCN* overexpression may be ascribed to indirect epigenetic dysregulations acting on a negative regulator of *MYCN* expression [104].

**Figure 3:**
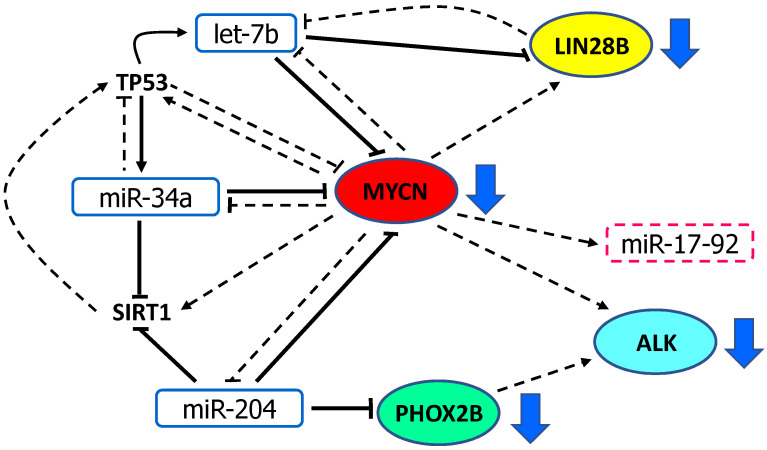
Physiological interactions between miR-34a, let-7b, miR-204, the transcription factors MYCN and PHOX2B and their target genes ALK and LIN28B through feedback and coherent feedforward loops. During physiologic regulation, miR-34a, let-7b and miR-204 exert effective downregulation of MYCN, PHOX2B and LIN28B (bold inhibitory lines) through feedback and coherent feedforward loops. In turn, MYCN and PHOX2B carry out a controlled activation of the transcription of their target genes (dashed arrows). The effect of MYCN activity over its targets and of LIN28B repression of let-7 biogenesis are also under control (dashed inhibitory lines). All three miRNAs are engaged with the TFs in feedback and coherent feedforward loops and the global effects are the reduced levels of all proteins encoded by target genes (large blue arrows).

**Figure 4:**
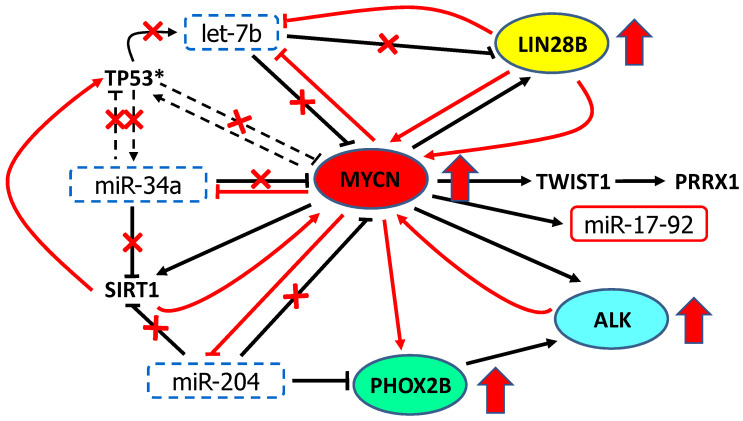
Disruption of physiologic regulation and generation of aberrant transcription activations and feedback loops between miR-34a, let-7b, miR-204, the transcription factors MYCN and PHOX2B and their target genes ALK and LIN28B in neuroblastoma. In NB, underexpression of miR-34a, let-7b and miR-204 (dashed boxes) affects their inhibitory functions (red crosses) and leads to increased levels of TFs and target genes. TF overexpression strongly activates target transcription (bold black arrows) with the consequent disruption of the physiologic regulation and feedback loops (red crosses), generation of enhanced transcription and aberrant feedback loops (red arrows and red inhibitory lines) with a global upregulation of target genes and increased levels of their encoded proteins (large red arrows). De-acetylated TP53 is indicated with an asterisk (TP53*).

In this review, we have focused on the regulatory circuitry linking TS miRNAs let-7, miR-34 and miR-204 with the oncogenic TFs MYCN and PHOX2B and their transcriptional targets *ALK* and *LIN28B*, and reconstructed their cross-talk. Physiologically, these miRNAs downmodulate *MYCN* and *PHOX2B* and, directly or indirectly, the expression of their targets *ALK* and *LIN28B* within a network of regulatory feedback and coherent feedforward loops (Figure 3) that are disrupted in NB, thereby generating a loss of inhibitory functions and aberrant transcription activations (Figure 4).

Below, we enxpand upon the role of these genetic determinants and their epigenetic perturbations, starting from their involvement in physiological development of NCC derivatives towards the pathogenetic dysregulation leading to NB oncogenesis.

### 5.1. miR-34

The role of miR-34 as a TS in oncogenic pathways is well characterized, but it also has important roles in neurodevelopmental and neuropathological processes.

During EMT, besides the activating functions of many TFs, p53 and a growing number of miRNAs have been identified as negative regulators. EMT-TFs and miRNAs, including miR-34, are often engaged in double-negative feedback loops forming switches that control the transitions from epithelial-to-mesenchymal cell states [105]. Well characterized is the miR-34-SNAI1 axis, whereby a miRNA and a TF use a double-negative feedback mechanism to repress each other to maintain cell homeostasis [70].

miR-34a has multiple developmental stage-specific activities, it promotes proliferation of NPCs, suppresses neuroblast migration, and regulates neurite outgrowth [106].

miR-34a is transcriptionally activated by all members of the TP53 family (TP53, TP63 and TP73) [107] and is an essential regulator of NPC differentiation via the suppression of hundreds of target genes, among which is the class III histone deacetylase SIRT1 (Sirtuin 1), a critical regulator of neuronal differentiation and survival [108]. Therefore, in differentiated neurons there is an intricate balance of miR-34a levels and SIRT1 levels/activity that maintains neuronal survival and function [107].

SIRT1 also regulates p53-dependent apoptosis through deacetylating and stabilizing p53. Due to a reduction in SIRT1 expression by miR-34a, an increase in the levels of acetylated p53 leads to the activation of p53 pro-apoptotic target proteins (i.e., the cyclin-dependent kinase regulator p21 and the p53-upregulated modulator of apoptosis, PUMA) [108]. Thereby, the miR-34a/SIRT1/p53 signaling pathway forms a positive feedback loop wherein p53 induces miR-34a and miR-34a activates p53 by inhibiting SIRT1, playing an important role in cell proliferation and apoptosis [109].

MYCN can directly induce the transcription of the class III histone deacetylase SIRT1 [110], which is downregulated by miR-34 (Figure 3).

Moreover, TP53 has been identified as a mediator of nerve growth factor (NGF) that induces the expression of miR-34a, which in turn contributes to neuronal differentiation and maintains the mature neurons in a non-proliferative stage by arresting cells in the G1 phase. It has also been shown that increased expression of miR-34a controls the TP53 level in a feedback inhibition manner, preventing differentiated cells from TP53-induced apoptosis [111].

A major influence of miR-34a on neurogenesis likely occurs through its multiple targets within the Notch signaling pathway and NPC impaired neurogenesis, which is likely due to the suppression of Numblike, an endocytic adaptor that negatively regulates Notch signaling, resulting in the suppressed expression of pro-neural gene products such as NeuroD1 and Ascl1 [112].

Another point of relevance to miR-34a regulation of NPC differentiation is its role as a TS in brain cancers like glioma and medulloblastoma [113,114] and also in NB via the targeting of multiple oncogenes such as *BCL2*, *MET*, *c-MYC*, *MYCN*, *CDK4*, *c-SRC* and *PD-L1* [35,36,37,90,91,92].

The lateral inhibition mechanism mediated by the Delta/Notch pathway plays a crucial role in the early phase of differentiation, where the delta-expressing cells give origin to the neuronal lineage, while the Notch-expressing cells remain undifferentiated or give origin to other lineages. miR-34 has been shown to inhibit Notch signaling, thus confirming its role in neuronal differentiation in a model of medulloblastoma [115].

MiR-34a was the first miRNA identified as a putative TS in NB for its ability to downregulate TFs and other genes essential for cell proliferation, particularly *MYCN* expression, through the direct binding to the 3′UTR of its mRNA [36,116] (Figure 3).

Interestingly, the gene encoding miR-34a maps to the 1p36 region [91], frequently deleted in NB tumors (25–30%) and associated with an aggressive phenotype [6].

miR-34a is generally expressed at lower levels in unfavorable primary NB tumors and cell lines [35,36,37] with a consequent increment of MYCN expression (Figure 4). To define the TS functions of miR-34a, several studies based on miR-34a replacement into NB cells have shown growth inhibition, induction of caspase-dependent apoptosis or differentiation induction, especially in NB cell lines with 1p36 hemizygous deletion [91] or with *MYCN* amplification [36,37,116]. Most importantly, other studies in vivo have demonstrated miR-34a as a potential therapeutic molecule by using synthetic mimics administered through lipid emulsion vehicles [117] or entrapped into nanoparticles. Specifically, evidence of miR-34a replacement as an effective therapy was provided for liver [118,119,120], prostate, lung [121] and for NB using untargeted [122] or targeted nanoparticles [123,124].

MiR-34a replacement in an orthotopic murine model of NB significantly reduces tumor growth, identifies novel effects on phospho-activation of key proteins involved with apoptosis and decreased angiogenesis [35]. More recently, further evidence has been provided to show that NB-targeted liposome delivery of miR-34a and let-7b mimics, alone and in combination, in orthotopic mouse models reduces cell division, proliferation, neo-angiogenesis, tumor growth and burden in a statistically significant manner [124] and improves mouse survival in pseudo-metastatic models. In the same study, miR-34a also induced apoptosis, in keeping with other reports [35,123], and more significantly in combination with let-7b [124].

The first compound based on miRNA mimics entered into a phase I clinical trial was MRX34, a liposomal miR-34 mimic compound tested in patients with liver cancer and other tumors [122]. Unfortunately, this trial was prematurely terminated because of severe immune-related events, mainly attributable to the untargeted liposome carrier [125], which emphasizes the need of tumor-targeted delivery systems for therapeutic approaches. Moreover, the use of miR-34 replacement in combination with chemotherapeutic agents has shown a synergistic effect [126,127].

It has also been demonstrated that PD-L1, a co-inhibitory factor of the immune response having a significant negative role in cancer progression when overexpressed, is regulated by TP53 via miR-34. PD-L1 downmodulation by miR-34 thus identifies a novel mechanism of tumor immune evasion regulated by the p53/miR-34/PD-L1 axis. [92].

### 5.2. Let-7

Lethal 7 (let-7) miRNA members belong to a well-known family of miRNAs known to regulate cell cycle, embryonic development, and maintenance of differentiated tissues where it is highly expressed, while it is under-expressed or deleted in various cancers leading, to increased cell division [128].

The global underexpression of the let-7 miRNA family observed in many cancers is affected by the RNA binding proteins LIN28A/B, which block let-7 biogenesis (Figure 3) and consequently the TS function of all let-7 family members [129]. LIN28A/B are also targets of the let-7 family, thus creating a double-negative feedback loop (Figure 3).

Let-7 miRNAs are essential for sympathetic neuroblast proliferation during normal development. LIN28B is highly expressed in developing tissues and sustains stem and progenitor cell identity by blocking the biogenesis and differentiation function of the let-7 miRNA family [33]. To clarify the role of LIN28A/B and let-7 during neurogenesis and NB development, a study was carried out in chick sympathetic ganglia, where LIN28A/B and let-7 resulted not only in undifferentiated progenitor cells, but also in proliferating noradrenergic neuroblasts. LIN28 knockdown decreases proliferation, whereas let-7 inhibition increases the proportion of neuroblasts in the cell cycle, indicating that proliferation was maintained by LIN28A/B and repressed by let-7 [33] (see LIN28 paragraph).

The decrease in let-7 miRNAs leads to overexpression of their oncogenic targets, including *KRAS*, *HRAS*, *HMGA2*, *BLIMP1*, *PD-L1*, *c-MYC*, *MYCN* and *LIN28B* [18,20,21,34,93,94,95,130,131,132] (Figure 4).

As let-7 target *c-MYC* and *MYCN* [131] and *LIN28B* is a transcriptional target of both *c-MYC* in multiple human and mouse tumor models [133] and *MYCN* in NB [20], the inhibition of let-7 is c-MYC/MYCN-mediated via LIN28. In NB, elevated LIN28B expression levels inhibits let-7 with consequent de-repression of *MYCN* [20] that reinforces cancer cell proliferation, activating the feedback loops of the MYCN-LIN28B axis [18,19,20,21] (Figure 4).

In tumor cells with underexpression of let-7, restoration of its normal level of expression by synthetic mimics was found to inhibit cancer growth by targeting various oncogenes and inhibiting key regulators of several mitogenic signaling pathways. Ectopic expression of let-7 in lung cancer cell lines altered cell cycle progression, reduced cell division and inhibited cellular proliferation in vitro and suppresses tumorigenesis in mouse models of lung cancer [93,121,128,132,134,135]. These functional effects are attributable to the ability of let-7 to down-regulate its target oncogenes, confirming that let-7 has an important role as a TS by direct or indirect repression of multiple genes involved in cell cycle and cell division functions and that it is a master regulator of cell proliferation pathways [93,94,136,137].

Furthermore, let-7b interferes with the proliferation and growth of primary malignant melanoma cells by targeting and suppressing important cell cycle molecules, such as cyclin D (CCND1) [138] and elevated let-7 expression levels inhibitsHMGA2 expression and suppresses the metastasis signaling cascade involving LIN28 and let-7 in breast cancer cells [139]. In breast and lung cancers, data also suggests that let-7 regulates apoptosis and cancer stem cell differentiation [140].

TP53 is also involved in the regulation of let-7 miRNA family members by remodeling the AGO2–miRNA–mRNA interaction. TP53 directly associates with AGO2 to induce or reduce loading of a subset of miRNAs, including let-7 (Figure 3), therefore, their cellular abundance or differential association with AGO2 are involved in an intricate network of regulatory feedback and feedforward circuitries [141].

Additionally, as let-7 post-transcriptionally downregulates PD-L1 expression, it has been demonstrated that the use of a LIN28 inhibitor increases let-7 levels and suppresses PD-L1 expression, leading to reactivation of antitumor immunity in vitro and in vivo [130].

A combinatorial supply of miR-34a and let-7b carried by neutral lipid emulsion or untargeted nanoparticles has been employed in lung cancer using transgenic mice bearing *KRAS*/*TP53* mutant non-small cell lung cancer (NSCLC) cells [117,121]. In a recent study, the delivery of liposomes entrapping miR-34a and let-7b in NB mouse models showed significant inhibition of cell division, proliferation, neo-angiogenesis, tumor growth and improved mouse survival [124]. These functional effects are mainly due to the replenishment of adequate levels of miR-34a and let-7b that restores their direct inhibitory regulation over *MYCN* and *LIN28B* and indirectly over *ALK* expression [124].

The potential use of let-7 as a chemo-sensitizer has also emerged in KRAS mutant NSCLC cells, in which let-7b repletion selectively sensitized KRAS mutant tumor cells to paclitaxel and gemcitabine by diminishing MEK/ERK and PI3K/AKT signaling [142]. Interestingly, in orthotopic NSCLC xenografts carrying mutant KRAS/TP53, let-7 and miR-34 have been supplied in combination with erlotinib, an EGFR tyrosine kinase inhibitor, demonstrating a significant synergism that improves treatment efficacy [126]. These data are promising in view of innovative therapeutic approaches for targetable mutations of KRAS that are enhanced in relapsed NB [1,143].

### 5.3. miR-204

In a Medaka fish model system, miR-204 has been demonstrated to be essential for a correct axonal extension of retinal ganglion cells to the brain [144].

MiR-204 is involved in several steps of the molecular control of NC development, including induction, migration and neuron or glia differentiation [67]. The *PHOX2B* gene has been demonstrated to be a target of miR-204 (Figure 3) that is able to recognize two binding sites in the proximal and distal regions of the *PHOX2B* 3′UTR [23,38], thus suggesting that miR-204 could also act in the early phases of neuronal differentiation by driving expression towards the glial lineages through the reduction of *PHOX2B* expression.

According to this hypothesis, miR-204 has been demonstrated to be a transcriptional target of Sox10 and to inhibit oligodendrocyte precursor cell (OPC) proliferation, thus inducing differentiation into mature olygodendrocytes [145]. Similarly, Sox10 could downregulate PHOX2B expression through miR-204 during the switch between the neuronal and glial fate of NCCs in sympathetic neuron differentiation. Moreover, miR-204 has been observed to be enriched in neuronal axons, where it drives neuronal guidance.

miR-204 is involved both in the fate of dopaminergic neurons and in the control of the maintenance of quiescent neuronal adult stem cells [146,147]. More widely, miR-204 is a non-coding RNA with opposite effects on differentiation depending on the tissues, i.e., it is an inhibitor of osteoblast maturation while it acts as an inducer of adipose cell differentiation [148]. Consequently, miR-204 plays a double role in cancer, acting as a TS miRNA or oncomiR depending on the tumor type [149].

**Figure 5:**
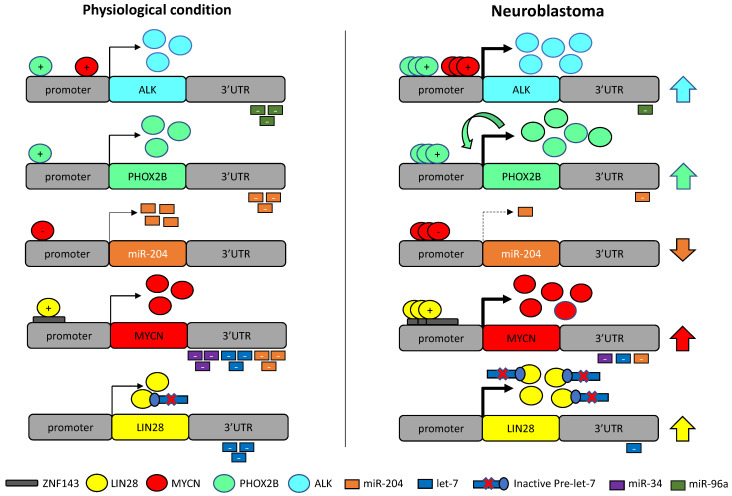
Transcriptional and post-transcriptional relationships between ALK, PHOX2B, MYCN, LIN28, miR-204, mir-34 and let-7. Genes are schematically represented by colored coding regions inserted between grey upstream (promoter) and downstream (3′UTR) regulatory regions. For each gene, transcription factors (circles) acting on its promoter and/or microRNAs (rectangles) acting on the 3′UTR, are shown in both physiological (**left**) and neuroblastoma (**right**) conditions. Thick or dashed arrows indicate an increased or decreased level of expression, respectively, of the gene in NB with respect to the physiological corresponding condition, in which gene expression level is represented by thin arrows. The protein products for each gene are shown as circles painted like the corresponding gene. The number of circles/rectangles represents the amount of product reflecting these levels of gene expression. Activation or inhibition effects of TFs are represented by “+” or “−”, respectively, within the circles on the promoter regions. In NB condition, the colored up and down arrows on the right indicate the resulting protein expression levels for each gene.

Global analyses of miRNA and mRNA expression profiles of tissues at different stages of tumorigenesis from TH-MYCN transgenic mouse model, have highlighted miRNA–mRNA interactions operating during NB oncogenesis. This study has demonstrated that miR-204 directly binds to the 3′UTR-mRNA of *MYCN* and inhibits *MYCN* expression (Figure 3). It has also revealed that MYCN binds to the miR-204 promoter to repress miR-204 transcription (Figure 5) and an increase in miR-204 expression was consistently observed in NB cells following MYCN silencing [39]. These findings disclose a double negative feedback loop between miR-204 and MYCN (Figure 4) by which, when overexpressed, MYCN self-perpetuates. Therefore, miR-204 has been identified as a TS miRNA in NB and MYCN-mediated repression of miR-204 transcription explains the low miR-204 expression in high grade MYCN amplified NB cases [39]. Consistently, miR-204 enforced expression significantly inhibits NB cell proliferation in vitro and tumorigenesis in vivo through the enhanced repression of MYCN expression. Although the starting event of this feedback loop is still to be identified, these observations add a novel autoregulatory network leading to NB cell growth [39].

In addition, miR-204 targets SIRT1 [150,151], reinforcing the inhibitory function of miR-34a over the gene (Figure 3) that is lost when miR-204 is under-expressed (Figure 4).

Moreover, it has been observed that miR-204 can act as an apoptosis inducer [152]. However, the miR-204 binding site in the 3′UTR of the anti-apoptotic B-cell lymphoma-2 (BCL2) contains the consensus sequence (GAA) for the RNA binding protein Tra2β, acting as activator of BCL-2 expression. Therefore, mir-204 and Tra2β compete for the regulation of BCL2 expression, as shown by an increased association between miR-204 and BCL2α 3′ UTR following *Tra2β* knockdown. Such competition can lead to opposite effects on cell survival as Tra2β regulates apoptosis by modulating Bcl-2 expression through its competition with miR-204 [153]. Moreover, low levels miR-204 could result in anti-apoptotic effects in NB—processes mediated by high levels BCL-2 [154].

Neurons derive from neural progenitors and the Delta-Notch signaling pathway plays a major role in these cell fate decisions. Interaction with ligands triggers a proteolytic processing of Notch receptors and the translocation of Notch intracellular domains (NICDs) to the nucleus, where they activate transcription of the effector genes Hairy and Enhancer of Split (HES) homologs [155]. In vivo studies have shown that Notch2 NICDs expressed in NSCs promote proliferation and prevent neuronal lineage entry, thus suggesting that Notch2 plays a role in NB pathogenesis; such a hypothesis is supported by the observation that miR-204 is a negative regulator of tumor invasion and its upregulation inhibits cancer cell proliferation by targeting Notch2 mRNA [156], as confirmed by the fact that depletion of miR-204 by BANCR-mediated sponging contributes to the growth and invasion of melanoma through Notch2 upregulation [157].

In addition, the Ephrin-B3 (EFNB3) gene has been demonstrated to be a direct target of miR-204 [144], although its significance in NB is unclear as *EFNB3* expression is directly correlated with a favorable NB prognosis, while in miR-204 downregulation is associated with opposite effects [40]. This apparent discordance could rely on the first observation in NB with normal expression of MYCN, while lower expression of miR-204 was detected in patients with known high-risk prognostic factors including MYCN amplification [40]. In accordance with these observations, the 3′ UTR of neurotrophin receptor NTRK2 (TrkB) gene, a high-risk prognostic marker involved in NB chemoresistance, is also a direct target of miR-204 [40].

All these results suggest that an impaired post-transcriptional regulation mediated by low levels of miR-204 plays a crucial role in NB development due to the consequent high expression of oncogenes normally downregulated during neural differentiation.

## 6. Key Transcription Factors and Target Genes in Neuroblastoma

### 6.1. MYCN and c-MYC

*MYCN* was identified in 1983 as an amplified gene homologous to the oncogene *v-myc* but distinct from *c-MYC* in human NB [158,159]. MYC oncoproteins, c-MYC, N-Myc or MYCN and L-Myc or MYCL, are TFs that are structurally very similar that regulate the expression of many target genes by activation or repression of transcription.

MYC transcription factors are master regulators of many processes in development, physiology and oncogenesis, including cell cycle entry, differentiation, survival, pluripotency, ribosome biogenesis and metabolism and coordinate a complex transcriptional response for cell growth and proliferation.

The first demonstrations that MYCN and MYC show powerful oncogenic activity were experimentally achieved in rat embryo fibroblasts where the enforced expression of these TFs promoted a transformation and induced proliferation and cell cycle progression [160].

Since the first studies in NB cell lines, it has been known that *MYCN* and *c-MYC* control their expression via auto-regulatory loops and via repressing each other at defined promoter sites [161]. NB cell lines with high expression of *MYCN*, because of the gene amplification, lack c-MYC expression, indicating that *MYCN* function could serve as a surrogate for c-*MYC* function.

The concept that MYC and MYCN can compensate for each other has been supported by many studies on development, as reviewed by Huang and Weiss [162]. As an example, mouse embryonic stem cells (ESCs) homozygous for deletion of either MYC or MYCN were shown to have normal morphology without aberrant proliferation or differentiation compared to wild-type ESCs [163]. Furthermore, in murine development, MYCN knocked-in at the MYC locus had the ability to rescue embryonic lethality associated with the loss of c-Myc and to restore immune functions in MYC knockout mice, although MYCN knocked-in animals were smaller and had few developmental defects [164]. However, even if MYCN knock-in can compensate for knockout of MYC, knockout of either MYC or MYCN resulted in embryonic lethality at E10.5–E11.5 [163], suggesting that in this period of embryonic development endogenous MYCN and MYC cannot completely compensate for each other, most likely for the distinct spatiotemporal expression patterns displayed by MYC family proteins [162].

This indicates a general functional similarity between these TFs in regulating certain lineages of murine cell growth and differentiation during embryogenesis and late development.

The expression of MYCN is neural-tissue specific and is highest in the forebrain, kidney, and hindbrain of newborn mice, while MYC expression is detected in a broad spectrum of tissues in newborn mice and markedly decreases in most tissues of adult mice, remaining at high levels only in adrenal and thymus tissues [165].

MYCN is highly expressed during fetal brain development [166] and NC-deriving lineages [77,167], while in brain and differentiated tissues of adult mice its expression is almost absent [162].

MYCN is initially expressed at high levels, thus promoting ventral migration of NCCs from the neural plate border [76], then its expression decreases at very low levels in migrating NCCs, to then be re-expressed in NC-derived lineages for maintenance of neural fate [77]. Conversely, *MYCN* is downregulated for terminal differentiation and functionality of sympathetic neurons [77].

*c-MYC* has been shown to contribute to the long-term maintenance and proliferation of the ESC phenotype and iPSCs in association with pluripotency genes such as *Nanog*, *Oct3/4*, *Sox2* and *Klf4* [168] that promote a specific miRNA expression program within the CRC controlling ESC identity [169]. Additionally, high N-myc levels have been shown to play a crucial role in the maintenance of pluripotency and self-renewal in murine ESCs and iPSCs by inducing the expression of some pluripotency genes, such as *lif*, *klf2*, *klf4* and *lin28b*. Remarkably, N-myc levels closely correlated with the expression of all these genes in NB and all but *lif* in neuronal progenitors [170], confirming the role of *MYCN* in the maintenance of pluripotency features also in cancer cells. Moreover, N-myc overexpression promotes the expansion of Phox2B-positive neuronal progenitors to drive NB development [79]. Conversely, the conditional loss of MYCN results in spontaneous differentiation, suggesting that MYCN needs to be downregulated for the terminal differentiation of neurons [77].

The paradigm by which *MYCN* expression is highest in immature cells in newborn mice, and it is dramatically reduced in differentiated adult tissues, is consistent with the finding that differentiation of NB cells is associated with reduced expression of *MYCN* [171].

Interestingly, *MYCN* is down-regulated during retinoic acid (RA)-induced differentiation of NB cell lines before the occurrence of cell cycle and morphological changes, confirming its direct role in blocking differentiation pathways [172,173].

Almost all MYCN-amplified NB cell lines are resistant to RA treatment due to the high oncogene expression that represses differentiation-promoting genes [162].

Upon MYCN overexpression in a MYCN-inducible NB cell line, several differentiation-relevant genes, including LMO4, CYP26A1, ASCL1, RET, FZD7 and DKK1, and a broad network of transcriptional regulators involved in regulating retinoid responsiveness, such as Neurotrophin, PI3K, Wnt and MAPK, have recently been identified [174]. This study has revealed that TGF-β signaling is a key regulator of the MYCN-mediated retinoid resistance. The existence of crosstalk between MYCN and TGF-β discloses a targetable vulnerability of the MYCN network that can be exploited for therapeutic co-targeting of the RA and TGF-β pathways [174].

Furthermore, MYCN-amplified NB cell lines resistant to RA treatment are, instead, sensitive to RNAi-mediated silencing of *MYCN*, suggesting that this novel therapeutic approach may be effective in *MYCN*-amplified NBs that have complete or partial resistance toward RA [175].

Among the pathways involved in the differential regulation of MYC proteins during embryonic organogenesis, Sonic hedgehog (Shh) signaling, which plays a key role in cancer and chemo-resistance, activates MYCN, while the Wnt/beta-catenin pathway, promoting stem cell regeneration and cell survival in cancer, activates c-MYC [176].

Deregulation in cancer often leads to constitutive overexpression of *MYC* genes, achieved through gross genetic abnormalities, including gene amplification; chromosomal translocations; increased enhancer activity, mainly through a mechanism of enhancer hijacking; and aberrant signal transduction leading to increased c-MYC/MYCN transcription or increased mRNA and protein stability [176,177,178].

Gene amplification is the most commonly observed alteration in cancer for the MYC gene family, and *MYCN* amplification is found particularly in NB [159] in about 25% of diagnosed cases and is strongly associated with poor prognosis, thereby being a defining feature of high-risk NB [2,6,10,179].

Amplified *MYCN* is consistently associated with high *MYCN* mRNA and protein levels. In fact, the high oncogene expression is predictive of poor outcome with or without the gene amplification [180,181].

MYCN activating mutations have not been described for a long time. However, within the Therapeutically Applicable Research to Generate Effective Treatments (TARGET) project, a rare somatic variant (1.7%) was described causing a p.Pro44Leu alteration that might be clinically relevant in case it confers MYC dependency [29].

Since the first experiments using ectopic *MYCN* expression in cell lines and targeted MYCN overexpression in peripheral NC transgenic NB mouse model, strong evidence that increased MYCN activity is involved in tumor initiation and progression of NB has been provided [160,182].

The generation of MYCN transgenic mouse models has greatly increased our knowledge about the molecular bases of NB pathogenesis. The transgenic expression of MYCN in the NC lineage of mice or zebrafish alone, or in combination with LMO1 or activated ALK (see paragraph ALK), develop MYCN-driven NB, establishing that overexpression of MYCN in migrating NCCs can initiate the disease [182,183,184,185].

Tumors in these models have a prolonged latency and showed recurrent chromosomal copy number abnormalities, suggesting that genetic mutations or deregulated expression of other genes in addition to dysregulated expression of MYCN may be required to promote neuroblast transformation.

To confirm that the redundancy of c-MYC and MYCN has an active role also in NB tumorigenesis, it has been demonstrated that augmented expression of c-MYC and/or MYCN proteins defines extremely aggressive MYC-driven NB [186,187,188].

Both MYCN and c-MYC play their oncogenic role by regulating the transcription of hundreds of TGs through directly binding to their promoters. Their enhanced activity contributes to almost every aspect of tumor formation: genomic instability, unrestricted cell proliferation, inhibition of differentiation, control of cell growth, cell cycle entry, angiogenesis, reduced cell adhesion, metastasis, metabolism, survival, apoptosis, pluripotency, self-renewal, immune surveillance, DNA replication and RNA synthesis [10,162,189,190,191,192].

MYCN can activate the transcription of a great number of TGs, among which *CDK4*, *CHK1*, *ID2*, *MCM*, *MYBL2* and *SKP2* promote cell proliferation, *MDM2* and *TRKB* sustain cell survival, *TWIST1* sustains cell survival and promotes EMT, genes encoding the cell cycle-regulated kinase AURKA promote tumor development and progression, FAK and integrins are involved in migration and metastasis spread, *BMI1* and *DLL3* hold self-renewal, *VEGF* boosts angiogenesis, *KLF2*, *KLF4*, and *SSEA-1* maintain pluripotency, as does *TP53* and consequent TP53-driven apoptosis [162].

MYCN also activates the transcription of two key oncogenes in NB, *ALK* [16] and *LIN28B* [20] (Figure 3) (see details in ALK and LIN28B paragraphs).

Additionally, MYCN can suppress the expression of TGs promoting differentiation (*CDKL5*, *TG2* and *P75NTR*), leading to cell cycle arrest (*DKK1*, *CCNG2*, *CDKN1A* and *TP53INP1*), immune surveillance (*MCP-1/CCL2*), the inhibition of migration and antagonizing metastasis (encoding E-cadherin, Integrins and *TIMP2*) and genes inhibiting angiogenesis (*INHBA* and *IL-6*) [162].

As MYCN can also activate apoptotic pathways via TP53 that inhibit tumor formation, this mechanism may underlie some spontaneous NB regression [193]. Nevertheless, because of activation/repression of the above-mentioned genes, the global effect is that MYCN firmly drives oncogenesis and the maintenance of a highly malignant phenotype and sustains progression and metastatic spread.

An enhanced activation of *MYCN* expression also broadly influences miRNA expression, thus leading to a significant over- or under-expression of specific miRNAs in *MYCN* amplified tumors relative to *MYCN* single copy tumors [32,98,194]. A major example of the direct upregulation of pro-tumorigenic miRNAs by MYCN is represented by the miR-17–92 cluster [195]. Yet, the activation of MYC TFs mainly leads to a widespread repression of miRNA expression, including potent TS miRNAs such as miR-34a and let-7 family members [196].

As previously cited, LIN28B is induced by c-MYC, leading to the c-MYC-mediated repression of let-7. This loss-of-function of LIN28B impairs MYC-driven proliferation [133]. Similarly, MYCN also regulates *LIN28B* expression via interaction with the *LIN28B* promoter and establishes a direct MYCN-LIN28B regulatory axis through which elevated LIN28B expression levels contribute to NB tumorigenesis via let-7 dependent de-repression of MYCN [20] and feedback loops that regulate the MYCN-LIN28B axis [18,19,20,21] (See LIN28B paragraph) (Figure 4).

*MYC* genes are also key players in therapeutic resistance, particularly as mediators of drug resistance or sensitivity in NB [197].

For example, altered MYCN influences cytotoxic drug response in NB via regulation of the multidrug resistance-associated protein (MRP) gene expression, so that MYCN overexpression increases MRP expression and the resistance specifically to MRP drug substrates, while downmodulation of MYCN lowers MRP expression and significantly increases sensitivity to the high affinity MRP substrates [198]. Indeed, this accurate modulation of drug resistance in NB is affected through the direct interaction of MYCN with E-box elements within the promoter of MRP1, activating its expression [199]. Additionally, overexpression of the murine double minute 2 (MDM2) gene in NB is relatively common and leads to the inhibition of TP53. It is also associated with other non-canonical p53-independent functions, including drug resistance and increased translation of MYCN and VEGF mRNA [200]. Conversely, it has been demonstrated that the reactivation of p53/miR-34a/MYCN axis modulates the sensitivity to cisplatin in NSCLC [201], a chemotherapeutic commonly used for NB treatment. As TP53 monitors the expression of miR-34a, which directly targets *MYCN* to sensitize NSCLC cells to cisplatin [201], the same pathway might also be involved in NB.

The coding regions of *c-MYC* and *MYCN* are highly homologous with long 5′ and 3′ untranslated regions (UTRs) and gene products have similar sizes (50–55 kDa). The N-terminal region can interact with co-activators or co-repressors and contains several domains conserved among the MYC family members, whereas the C-terminal contains basic region/helix-loop-helix/leucine zipper (BR/HLH/LZ) motifs for the dimerization with the obligate partner MAX [192,202] for interaction with DNA and transcriptional activities. The MYC–MAX heterodimer binds to DNA consensus core binding sites 5′-CACGTG-3′ or other E-box variants with high affinity at the promoter of TGs [176] (Figure 5).

MYC proteins, which can only bind to regions of open and accessible chromatin, when present at deregulated high levels saturate the cis-regulatory landscape and can bind degenerate, non-canonical E-boxes with lower affinity (i.e., 5′-CANNTG-3′) proximal to promoters or at distant enhancers, leading to a constitutive transcriptional activation of TGs and pleiotropic transcriptional consequences [7,203], extending the MYC target repertoire. Notably, a previous study showed a predominance for the CATGTG motif in the MYCN amplified state and that MYCN binding is highly enriched in genomic regions of DNA hypermethylation [204]. As this co-localization is prominent within intra- and intergenic regions in addition to promoter sequences, these results suggest a possible dual role for MYCN and DNA hypermethylation, namely that of a classical transcriptional repressor of upstream genes and that of a mediator of global chromatin structure [204].

The levels of MYC binding at TGs are differentially modulated by additional co-factors, activating the MYC transcriptional program, even without its dimerization partner MAX [205]. Moreover, MYC proteins can also repress target genes mainly by a mechanism independent of the E-box binding that involves a complex formation with the cofactor Miz-1, the MIZ-1/Myc, which is able to promote the stabilization of Myc by inhibiting its ubiquitination and degradation [206]. Alternatively, MYC proteins can bind the zinc-finger protein SP1 to form the complex SP1/MYCN followed by the recruitment of the histone deacetylase HDAC1 that allows for the compacting of chromatin and transcriptional repression [206] or by the recruitment of SIRT1 to repress the transcription of MKP3 that leads to phosphorylation of the ERK protein, which in turn phosphorylates the MYCN protein with its consequent stabilization, thus creating a positive feedback loop between SIRT1 and MYCN [110] (Figure 4).

Another important aspect that should be considered is that MYC TFs are gene-specific and global gene-control factors, with a number of genes that rapidly respond to perturbations in MYC levels and other functional responses based on the cell chromatin landscape and gene expression program [7]. Independently of their activity in target gene expression changes, MYC proteins promote cell proliferation and tumorigenesis by altering basic transcription mechanisms [207].

As introduced above, the identification of two SEs and related TFs has defined specific transcriptional CRCs that underlie two distinct cell states in NB: an adrenergic-committed state, ADRN, characterized by a set of TFs including PHOX2A, PHOX2B, ASCL1, HAND2, GATA2, GATA3, MEIS2, LMO1, TBX2, ISL1 and many others, and an undifferentiated mesenchymal state, MES, defined by PRRX1, TWIST1, SNAI2, MAML3, NOTCH members, AP-1 TF family members (including JUN and FOS family members), and many others [41,42,50,52,55].

Kinetic studies of MYCN activation in NB have been performed to identify tumor-specific dependencies among CRC TFs, drawing the first dynamic chromatin and transcriptional landscape of MYCN perturbation in NB [7,51].

A genome-wide ChIP-Seq analysis detected a small number of transcription factors such as MYCN, HAND2, ISL1, PHOX2B, GATA3 and TBX2 belonging to the ADRN CRC that are essential for cell state and survival and represent dependencies in MYCN-amplified NB [51]. The authors demonstrated that each of these TFs is able to directly regulate the expression of its own gene as well as those encoding the other CRC TFs. Interestingly, the knock-down of only one member of the CRC can induce a decrease in the expression of several other members. As high-level expression of MYC or MYCN is also in *MYCN* non-amplified NBs, a similar set of TFs was hypothesized to form a CRC also in these tumors [51]. Recent evidence has emerged suggesting that local enhancers may be required for proto-oncogene expression on amplicons [208]. It has been demonstrated that a mechanism of enhancer hijacking is used in NB to activate c-*MYC*/*MYCN* expression [177,178]. Enhancer hijacking is a tricky mechanism by which, following structural rearrangements, translocations or other processes, distal regulatory elements, such as enhancers or SEs, may be brought into the proximity of other genes, thereby activating their transcription. In the case of *MYCN*, it has been shown that ectopic enhancers or SEs controlled by a specific CRC are juxtaposed to *MYCN*, highlighting the relevance of the CRC in driving *MYCN* oncogene overexpression [177]. A common set of ADRN CRC-driven enhancers has been found uniquely in *MYCN* expressing NB cells, indicating that *MYCN* expression is regulated by the CRC TFs, even in the context of gene amplification, in which ectopic enhancers or SEs are placed next to *MYCN* on amplicons [177]. This could mechanistically explain the previous observation that genetic depletion of CRC TFs represses *MYCN* expression even in *MYCN*-amplified cells [51].

MYCN acts as a global amplifier of transcription in NB, and it associates with E-box binding motifs in an affinity-dependent manner, binding to strong canonical E-boxes at promoters and, when overexpressed, invading weaker non-canonical E-boxes clustered at enhancers or other regulatory sites of the TGs.

**Figure 6:**
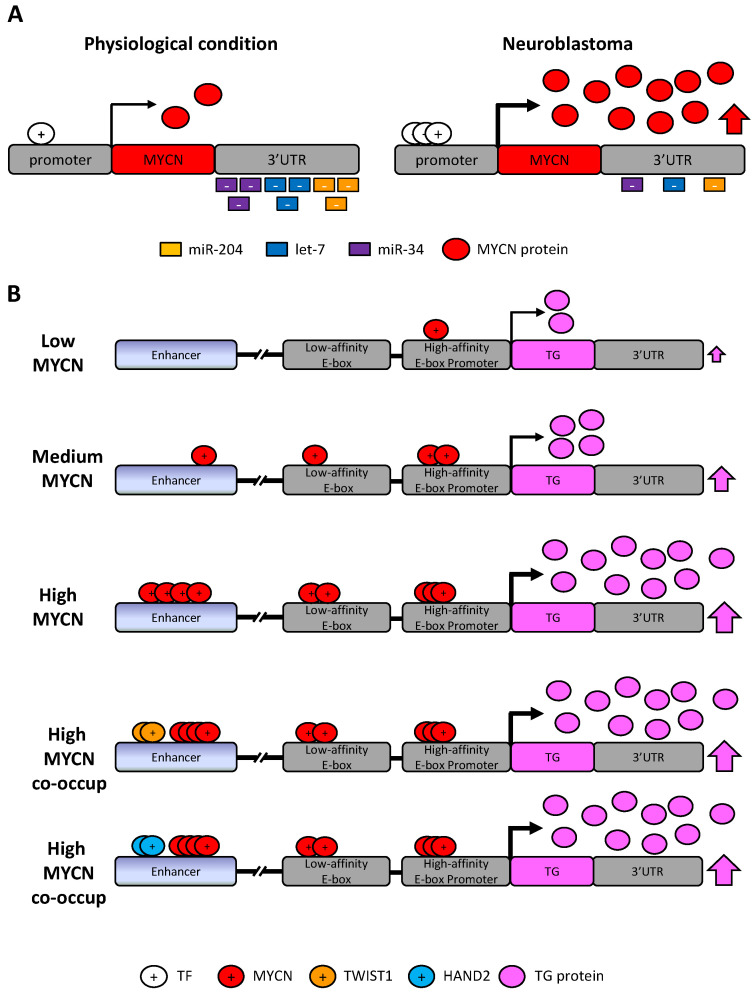
Schematic illustration of MYCN enhancer invasion/co-occupancy effects. (**A**) The two images show regulation of MYCN expression by a general activating TF (“+” white circle) in both physiological and NB conditions, where MYCN is amplified/overexpressed. The thick arrow indicates an increased level of MYCN expression in NB with respect to physiological conditions. The number of circles represents the amount of protein products, reflecting the levels of gene expression. While under physiological conditions there are adequate levels of TS microRNAs (rectangles), low TS miRNA levels are present in NB. (**B**) From the 5′ to the 3′end, a general MYCN target gene (TG) is represented by its enhancer (faded blue), high and low affinity promoters with E-box (grey), coding region (pink) and 3′UTR (grey). Circles represent a general TF (white), TWIST1 (yellow), HAND2 (cyan) and MYCN (red). The different levels of TG products are shown as consequences of low, medium and high MYCN levels, the latter in absence and the presence of additional TFs co-occupying the TG enhancer. “+” within circles indicate activating effects of TFs. The number of pink circles represents the amount of TG products reflecting levels of gene expression. Pink arrows on the right indicate the resulting level of TG expression for each condition.

The mechanism of enhancer invasion occurs because abundant MYCN proteins occupy the enhancers of TGs, frequently together with other TFs such as LMO1 [55,185,209], TWIST1 or HAND2 [7] (Figure 6).

From ChIP-Seq analysis, the lineage-specific TFs TWIST1 and HAND2, having a well-established role in promoting tumorigenesis, were retrieved across the NB cell lines of both states analyzed. As bHLH TFs recognize E-box CANNTG motifs and high-affinity TWIST1 and HAND2 sites were predicted at >80% of all MYCN enhancer sites, it was demonstrated that the clustered non-canonical E-boxes at enhancers invaded by MYCN were proximally occupied by TWIST1 or HAND2, contributing to driving oncogenic enhancer-driven transcription [7] (Figure 6).

The discovery that TWIST1 co-occupies enhancers with MYCN and is required for the expression of the MYCN enhancer axis thus result in MYCN-dependent proliferation [7]. TWIST1, which is a regulator of the mesenchymal lineage and of the dedifferentiated MES cell state in NB [41,42], is a transcriptional target of both MYCN and MYC [210]. TWIST1 specifies TGs but relies on other cues, including potentially MYCN, to enforce transcriptional activation or repression, thereby it can be considered a deregulated MYCN-specific NB dependency [7].

**Figure 7:**
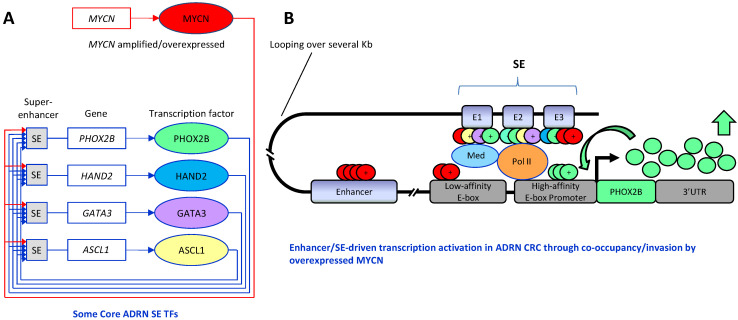
Enhancer/super-enhancer-driven transcription of ADRN CRC TFs by overexpressed MYCN. (**A**) Schematic representation of the ADRN CRC composed of a set of super-enhancer (SE)-associated lineage TFs (i.e., PHOX2B, GATA3, HAND2, ASCL1) co-occupied/invaded by MYCN (red arrows) following *MYCN* amplification/overexpression. The TFs (ovals) binds each other’s SE and induce a powerful feed-forward loop over all the other genes. (**B**) Structural drawing of the molecular interactions within the ADNR CRC: the *PHOX2B* gene is given as an example. Starting from the 5′ end, PHOX2B SE consists of three E-boxes, bound by all TFs taking part in the CRC and by MYCN. In addition, overexpressed MYCN activates the PHOX2B enhancer and PHOX2B positively self-regulates its transcription by interacting with its own promoter in a positive autoregulatory loop.

MYCN amplified or overexpressed under the control of an enhancer or a SE from a specific CRC through a mechanism of enhancer hijacking, in turn, highly activates the transcription of its direct TGs, but also of TFs belonging to the ADRN CRC (Figure 7A).

At a structural level, SEs are composed of the clustering of multiple enhancers in close genomic proximity of each other that, thanks to the recruitment of the mediator (Med) complex, interact with the basal transcription machinery and RNA polymerase II (Pol II) at promoters of target genes by a looping process [64]. We illustrate, as an example, how overexpressed MYCN is able to co-occupy/invade either enhancers or SEs in ADRN CRC (Figure 7A), leading to enhancer/SE-driven transcription activation of PHOX2B (Figure 7B) and of the other TFs belonging to the ADRN CRC. Likewise, overexpressed MYCN can co-occupy/invade either enhancers or SEs in MES CRC and lead to enhancer/SE-driven transcription activation, for example of TWIST1, and of the other TFs belonging to the MES CRC.

Cellular processes and developmental transitions are regulated by cell-type-specific enhancers.

The observed enhancer invasion by deregulated MYCN suggests that MYC family TFs may act through pre-established enhancers to amplify tissue-specific gene expression [211]. MYCN load at promoters and proximal enhancers predicts transcriptional responsiveness to MYCN shutdown. In fact, the loss of MYCN leads to a global reduction in transcription, more evident at the MYCN target genes with the highest enhancer occupancy [55]. These highly occupied MYCN TGs show tissue-specific expression and are linked to poor patient survival [7].

### 6.2. PHOX2B

The paired-like homeobox 2B (*PHOX2B*) gene is a master gene of the SNS development and an essential regulator of the development and differentiation of NC derivatives. In fact, its absence leads to the complete loss of peripheral autonomic neurons due to a lack of expression of all the other TFs specific to the SNS [75].

The *PHOX2B* gene is involved through different pathogenic mechanisms in NB development, as is expected because of its role in the early steps of sympathetic neuron differentiation.

In particular, three kinds of *PHOX2B* genetic elements seem to play a role in NB occurrence: missense (MS) mutations, frameshift (FS) mutations and unbalanced gene expression found in both sporadic and familiar cases of isolated NB and in both isolated and syndromic forms of the tumor [28,212].

We have recently reported that mutations associated with isolated NB are clustered in two groups: MS mutations, which mostly arise in exon 1, and FS mutations, occurring in both exon 1 and exon 3, mainly localized upstream of the 20 polyAla region. Conversely, among PHOX2B mutations associated with syndromic NB, in which NB occur together with other neurocristopathies such as congenital central hypoventilation syndrome (CCHS) and/or Hirschsprung disease (HSCR), MS variants mainly hit the homeodomain (HD) while FS mutations can arise throughout exon 3 [23] (Figure 5).

Overall, syndromic NB is mainly associated with mutations in the sequence encoding the HD and in exon 3. FS mutations in both sporadic and syndromic NB arise downstream of the HD, thus suggesting that FS and MS do not share the same pathogenic mechanism. In particular, while MS mutations seem to disrupt the ability to bind a target promoter, thus decreasing PHOX2B-mediated genes’ transcription ability [213,214], FS variants appear to be predisposed to NB by different mechanisms. Among FS mutations, the “frame 2” subset, leading to elongated PHOX2B proteins due to stop codon loss, is associated with NB while “frame 3”, leading to truncated proteins, is mostly found in isolated CCHS [24]. These *PHOX2B* mutations, mostly found in NB, likely act by recruiting aberrant interacting proteins or by loosening proteins that regularly bind to PHOX2B. Alternatively, the aberrant C-terminal region could drive PHOX2B on non-physiological targets, thus inducing aberrant downstream gene expression.

Functional studies have demonstrated that both *PHOX2B* mutations associated with NB impair differentiation of progenitor cells towards the neuronal lineage, thus suggesting a dominant negative effect of mutant *PHOX2B* alleles on the WT protein [215]. In accordance, PHOX2B mutations associated with syndromic NB have been reported to revert the inhibitory effect of PHOX2B on SOX10 expression, whose expression should be mutually exclusive in the late differentiation stages of the autonomic nervous system, by inducing SOX10 expression and thus driving progenitor cells toward a glial differentiation [216].

In NB cells, PHOX2B and MSX1 expression are inversely correlated; such am observation is in accordance with the role of MSX1 in the activation of the NOTCH3 expression; in particular, NOTCH 3 is highly expressed in benign ganglioneuroma but lowly expressed in severe neuroblastoma, the latter being characterized by high PHOX2B expression [217].

Taken together, all this evidence suggests that, rather than definitively switching neurons to glia, PHOX2B mutations in NB can act by impairing neuronal differentiation and thus making cells susceptible to secondary transforming events.

*PHOX2B* overexpression has been detected in both NB cell lines and in NB patients [218,219]. PHOX2B levels, together with CRMP1, DBH, DDC, GAP43, ISL1 and TH, belong to the gene expression signature used to predict the NB outcome [220], whereby high levels of *PHOX2B* correlate with worse prognosis [221].

Interestingly, as we have demonstrated that PHOX2B drives *ALK* gene transcription (Figure 3) by directly binding its promoter, this regulatory relationship explains the co-overexpression of the two genes found in NB samples and NB cell lines [26,219,222] due to the downstream effect of *PHOX2B* overexpression in enhancing *ALK* transcription, underlying their functional cooperation in initiating and worsening NB pathogenesis.

PHOX2B expression is modulated at transcriptional and post-transcriptional levels.

At the transcriptional level, PHOX2B is positively regulated by itself in an autoregulatory loop that, if dysregulated, could underlie *PHOX2B* overexpression in NB [214,223].

*PHOX2A*, a paralogous gene of *PHOX2B* with an identical homeodomain, is both a direct target and a positive transcriptional regulator of *PHOX2B* transcription and its overexpression in NB cells may lead to *PHOX2A* and *PHOX2B* co-overexpression [219,224].

Moreover, it has also been reported that the NFκB and AP-1 pathways are involved in PHOX2B transcriptional regulation [225]. In particular, NFκB signaling is required for neural stem cells’ (NSC) early differentiation and inhibition of NFκB and in transgenic mice has been observed to induce the accumulation of Nestin, Sox2 and glial fibrillary acidic protein, thus maintaining NSCs’ quiescent [226].

At the post-transcriptional level *PHOX2B* expression is regulated by miR-204 (Figure 5), whose levels inversely correlate with NB prognosis, thus suggesting that the miR-204-PHOX2B axis is involved in *PHOX2B* overexpression in NB [38] (Figure 4). Specifically, by a luciferase reporter approach in NB cells, consisting in the expression of the firefly luciferase whose stability depends on 3′UTR PHOX2B, starting from the end of the coding region, a proximal and a distal site recognized by miR-204 have been identified [38,227].

In addition, single nucleotide polymorphisms’ (SNPs) genotyping of 11 NB cell lines showed three different *PHOX2B* haplotypes in the 3′UTR of the gene associated with different PHOX2B gene expression levels, likely interfering with its mRNA stability. Going into detail, it has been demonstrated that the SNP rs1063611 c*1455T > A is able to modulate the miR-204 effect of the pre-existing proximal site [38] and the rs114290493 c*161G > A has been shown to create ex novo a novel miR-204 recognition site on 3′UTR PHOX2B, which is absent in the presence of the G allele, that leads to luciferase mRNA degradation [227]. In accordance with high *PHOX2B* expression in NB in all NB cell lines analyzed, only the as rs114290493 “G” allele, associated with no miR-204 effect, was detected [38].

### 6.3. ALK

The Anaplastic lymphoma kinase (ALK) gene, originally identified as part of the chimeric nucleophosmin-ALK (NPM-ALK) protein in a chromosomal rearrangement associated with anaplastic large cell lymphoma [228], maps to chromosome 2p23 and encodes a tyrosine kinase receptor normally expressed at high levels in developing central and peripheral nervous systems districts, such as thalamic nuclei, spinal cord motoneurons and sympathetic, enteric ganglia and motor nuclei of the brainstem [229,230,231,232]. Its expression has been detected in sympathetic neuroblasts at E12.5 and E13.5 [44] and it seems to act by inducing neurogenesis in sympathetic ganglia [233,234]. However, although its role in the mammalian brain development is still not completely understood, in vivo experiments using zebrafish showed that ALK is essential for the development of the central nervous system as *alk* depletion by the morpholino approach revealed a severely compromised neuronal differentiation and neuron survival in the CNS [235].

Accordingly with these observations, transient ALK inactivation at embryonic stages induced long-lasting defects in the adult mouse brain, such as impaired neuronal connectivity and cognition, in addition to impaired neuronal migration and reduced neuronal proliferation of embryonic progenitor cells during development of the nervous system [236].

In particular, scRNA-seq experiments on cerebral organoids, treated with the ALK inhibitor ceritinib revealed differently expressed genes in radial glial cells (RGCs) and neural progenitor cells (NPCs) with altered hedgehog and hippo signaling [236].

The crucial role of ALK in neurodevelopment demonstrated was thus consistent with the successive identification of mutations in sporadic and familial cases of NB [11,12,13,237]. Particularly, missense and truncated mutating mutations, leading to a constitutive kinase activity activation of the receptor, have been identified in hot spots of the ALK coding region [12], mainly represented by the positions 1174 (p.F1174L), 1275 (p.R1275Q) and 1245 (p.F1245L), which account for 85% of all mutations in NB [238].

Interestingly, rearrangements and gains at 2p that occur in NB may affect *MYCN*, *ALK*, and *ALKAL2* genes, which are close to each other [239].

Similarly to PHOX2B, in addition to causative mutations, also ALK overexpression plays a pathogenetic role in NB. However, gene amplification accounts for a small percentage (about 5%) of ALK overexpressing NB cells [240,241], thus suggesting that ALK overexpression can also be ascribed to other mechanisms.

Promoter analysis has revealed that *ALK* is a direct target of MYCN (Figure 5), thus explaining why ALK expression is significantly high in NBs with amplified MYCN [16] (Figure 4). The induction of *ALK* expression was also observed in non-NB cells, suggesting that the transcription of the *ALK* gene is generally regulated by MYCN [16].

On the other hand, a luciferase assay of the ALK-expressing doxycycline-inducible system (either wt ALK or mutated ALK) in PC12 and NB cell lines revealed that activation of ALK results in the increased initiation of transcription activity in the *MYCN* promoter region, suggesting MYCN as a putative downstream target of ALK-mediated signaling [16].

In another study, it has been shown that the ALK regulatory axis includes a positive feedback loop, in which ALK stimulates an ERK5-mediated MYCN transcription [242] (Figure 4) through the PI3K, AKT, MEK3 and MEK5 signaling pathways, thus potentiating the oncogenic activity of MYCN. These results provide a possible explanation for the poor clinical outcome observed when *MYCN* is amplified together with activated ALK [243].

Promoter analysis has revealed that ALK is also a direct transcriptional target of PHOX2B [222] (Figure 5). This finding explains why these two genes have been found overexpressed in the great majority of primary NB samples and cell lines tested and the striking correlation between the transcription levels of ALK and PHOX2B (Figure 4) and its direct target PHOX2A [222].

In addition, low levels of miR-96 in neuroblastoma seem to play a role in *ALK* overexpression, as the ALK 3′UTR has been reported to be a target of miR-96 [102] (Figure 5).

Other than the presence of mutations and/or over-expression of *ALK*, further genomic rearrangements leading to ALK variants have been detected in NB that, together with gene amplification, are associated with very poor prognosis [238], thus conferring to ALK a gain of function role to ALK defects in NB pathogenesis.

Experiments of over-expression and knockdown of *ALK* in NB cell lines have demonstrated its role in tumor initiation and cell proliferation [11,12,13,16,237], angiogenesis [15] migration and invasion [16].

The role of the activating mutation of *ALK* in the tumorigenesis of NB has been extensively studied in transgenic animal models in association or not with the enforced expression of *MYCN*.

In a transgenic zebrafish model of NB in which MYCN-induced tumors arise from a subpopulation of neuroblasts, co-expression of activated *ALK* with *MYCN* triples the disease penetrance and markedly accelerates tumor onset by providing pro-survival signals that block the physiological apoptotic response and allowing for continued expansion and oncogenic transformation [184].

In mouse models, following the overexpression of ALK (p.F1174L) or MYCN or both in NC cells, the co-expression of these two oncogenes has led to the development of NBs with an earlier onset, higher penetrance, and enhanced lethality compared to those generated by the overexpression of the two oncogenes alone. These findings indicate that the combination of mutated ALK and MYCN signaling is sufficient to induce NB from sympathoadrenal progenitors and that ALK (p.F1174L) mutation potentiates the oncogenic activity of MYCN in NB [244].

In another study, using JoMa1, a murine multipotent NC progenitor cell line, immortalized with Tamoxifen-inducible Myc-ER^T^, it has been demonstrated that NC progenitor cells give rise to NB in vivo upon transformation with enforced expression of MYCN or ALK (p.F1174L) [245].

Expression of MYCN or ALKp.F1174L enabled these cells to grow independently of c-MycERT activity in vitro and caused the formation of NB-like tumors in vivo in contrast to parental JoMa1 cells, indicating that NBs occur as their malignant progeny [245].

To clarify the role of other activating *ALK* mutations or *ALK*-wt overexpression in NB tumor initiation, another study was conducted using the same inducible Myc-ER^T^ system in JoMa1 cells [246]. ALK-wt expression, like ALK-F1174L or ALK-R1275Q, in the absence of exogenous Myc-ER^T^ activity, was sufficient to induce the formation of aggressive and undifferentiated NC cell-derived tumors [246]. Therefore, ALK-wt overexpression and activating ALK mutants may not only upregulate MYCN mRNA expression, as shown in neuronal and NB cells [243], but also cooperate with MYCN in NB tumorigenesis [184,243,244,245,247] and they may also upregulate and cooperate with Myc, as observed in murine NCPC [246].

*MYCN* overexpression combined with activated ALK is sufficient to induce NB development in mouse sympathoadrenal cells, resulting in the fully penetrant and rapid generation of NB without any additional genomic alterations. Further evidence that MYCN cooperates with the mutational activation of ALK has been provided in chick sympathetic neuroblasts by a study aimed at evaluating the normal function of *MYCN* and *MYC* in the control of neuroblast proliferation and at examining the effects of overexpression of *MYCN*, *MYC*, and activated ALK, alone and in combination [248]. The authors demonstrated that *MYC* and *MYCN* overexpression elicits increased neuroblast proliferation that is maintained when *MYCN* and *ALKF1174L* are co-expressed. Proliferating *MYCN/ALKF1174L* neuroblasts display a differentiated phenotype but differ from *ALK*-expressing neurons by the upregulation of *SKP2*, *CCNA2*, *E2F8*, and *DKC1*. As inhibition of the ubiquitin ligase SKP2 reduces neuroblast proliferation, *SKP2* has been considered essential for maintaining the proliferation of *MYCN/ALKF1174L* neuroblasts [248].

Taken together, these results show that MYCN enforced expression with activated ALK cooperation leads to neuroblast proliferation and survival that may represent the initial steps toward NB development.

Following the discovery of *ALK* activating mutations in ∼8% of NBs, ALK has emerged as a tractable molecular target to counteract NB progression and several therapies aimed at inhibiting its tyrosine-kinase activity have been developed [249]. This has opened the possibility of further improving outcomes for the subset of patients with NBs carrying ALK variants by therapeutic approaches that include ALK inhibitors. In particular, several clinical trials using crizotinib have been established [250] and a phase III trial (https://clinicaltrials.gov/ct2/show/NCT03126916, accessed on 2 November 2021) is currently recruiting high-risk patients to study the effects of combining crizotinib with standard therapies. Unfortunately, the *ALK* F1174L mutation is associated with intrinsic and acquired resistance to crizotinib and co-segregates with MYCN in NB [244]. Therefore, further small-molecule inhibitors of ALK have been tested, of which crizotinib, ceritinib, alectinib, brigatinib and lorlatinib have been approved by the FDA [249].

In subsets of ALK mutated NBs, ceritinib has shown a complete clinical remission of both primary tumors and metastasis after 21 months of treatment [251]. In cellular and xenograft models expressing activated ALK, the concurrent inhibition of MDM2 and ALK was sufficient to overcome ceritinib resistance caused by MYCN amplification, thus suggesting that ceritinib might be more effective in combination with other therapeutic strategies [252]. Lorlatinib, which belongs to the third generation of ALK inhibitors, has been shown to inhibit most ALK mutants resistant to previous generation ALK inhibitors, including the *ALK* F1174L-mutated NB. Unfortunately, novel bypass mechanisms of resistance adopted by cells are also emerging against lorlatinib [253].

From a conceptual point of view, RNAi-based therapeutic approaches to silence *ALK* expression are more effective with respect to ALK inhibitors, because they rely on the design of specific siRNAs for either wt or the mutated gene transcripts that ensures an extremely high sequence-specificity. Nevertheless, for the systemic administration of RNAi molecules, the main challenge remains the delivery system and to date the knockdown of *ALK* expression has been successfully employed in pre-clinical models of NB only using NB-targeted liposomes to entrap *ALK*-directed siRNAs, either alone [15,254] or in combination with an ALK inhibitor [255].

### 6.4. LIN28B

LIN28A/B are RNA binding proteins that negatively regulate let-7 biogenesis (Figure 3) and act as proto-oncogenes responsible for the post-transcriptional downregulation of let-7 observed in many cancers. LIN28A bind the terminal loop of precursor let-7 and recruits the Terminal Uridylyl Transferase ZCCHC11 that polyuridylates the microRNA precursors, pre-let-7, thereby blocking let-7 biogenesis and their TS function. For LIN28B, the precise mechanism responsible for let-7 inhibition remains controversial [129]. However, the decrease in let-7 microRNAs due to elevated levels of LIN28A/B leads to overexpression of their oncogenic targets such as *MYCN* and many others [136].

LIN28s have been shown to influence development, metabolism, tissue repair, and disease processes in a manner that can be either dependent or independent of their effects on let-7 processing [33].

LIN28A/B and let-7 are essential for sympathetic neuroblast proliferation during normal development. LIN28B is highly expressed in developing tissues and sustains stem and progenitor cell identity by blocking the biogenesis and differentiation function of let-7 microRNA family, while it is normally down-regulated upon cell differentiation [33].

LIN28B is aberrantly upregulated and activated in NB by gene amplification and/or overexpression and is associated with poor patient survival, representing an independent risk factor for adverse outcomes [19].

Amplifications of the 6q21 region, including the *LIN28B* gene, occur at a low frequency and activating mutations or structural aberrations in the *LIN28B* coding sequence is very rare in NB [18], and only a genetic variant rs17065417, correlated with gene expression, has been identified in NB cell lines [256].

Enforced expression of LIN28B in embryonic mouse sympathoadrenal neuroblasts reduces let-7 levels, resulting in a strong increase in the amount of MYCN protein and also contributing to the stabilization of endogenous MYCN. MYCN in turn drives tumor growth, as is shown by reduced tumor size upon treatment with the MYCN antagonist JQ-1 [18], and elicits postnatal NB formation. Maintained proliferation of mouse JoMa1 neuroblasts in response to LIN28B overexpression suggests that NB evolves from the expansion of neuroblasts that fail to leave the cell cycle [18].

However, the normal function of LIN28B in the development of sympathetic neurons and chromaffin cells, the timing and the mechanisms involved in LIN28B-induced tumor formation are not completely known.

As it has already been mentioned in the paragraph dedicated to let-7, a study was conducted in chick sympathetic ganglia, where LIN28A/B and let-7 resulted expressed in either undifferentiated progenitor cells or in proliferating noradrenergic neuroblasts. In cultured chick, sympathetic neuroblasts LIN28A/B knockdown decreased proliferation, while let-7 inhibition increased the proportion of neuroblasts in the cell cycle, confirming that proliferation was sustained by LIN28A/B and repressed by let-7. However, LIN28B overexpression enhanced proliferation only during a short developmental period and later it did not reduce let-7 levels. These observations were confirmed in a mouse model with enforced expression of LIN28B, suggesting let-7-independent functions during initial development. Therefore, LIN28B-induced NB formation seems to require cooperation with additional signals activated in tumor founder cells at late postnatal stages [33].

Recent studies have highlighted the importance of zebrafish and xenopus models to better characterize the role of LIN28B during the early stages of NB development [257,258]. Such studies demonstrated that LIN28B overexpression in both vertebrate models support NB onset by inhibiting sympathoadrenal cell differentiation and by impacting NCC migration, particularly by increasing the migratory capacity of trunk NCC. By using a zebrafish transgenic model with overexpression of human LIN28B in the precursors of the parasympathetic nervous system, the authors demonstrate the pro-tumorigenic effects of LIN28B leading to the formation of NB-like tumors in 6-month old transgenic zebrafish. Tumor masses arising in these transgenic animals were histologically, immunohistochemically and ultrastructurally very similar to those arising in humans, being localized in the interrenal gland (the counterpart of the adrenal gland of mammals, a common site of NB onset) and expressing NB markers (such as TH, HuC/D, and synapthophysin) [258]. Interestingly, such data also suggest that LIN28B is able to support NB onset through let-7a-dependent and -independent mechanisms.

As mentioned before, *LIN28B* expression is induced by the *c-MYC* oncogene in multiple cancer models [133], while LIN28B is transcriptionally activated by *MYCN* in NB [20].

Upregulation of LIN28B in NB blocks let-7 precursors from being processed to mature let-7 miRNAs and shows genomic aberrations and extensive overexpression in high-risk NBs compared to several other tumor entities and normal tissues. Let-7 family miRNAs themselves are known to target *LIN28B* [137] (Figure 3), thus creating an autoregulatory feed-forward loop due to the commitment of NC cells during development and in NB (Figure 4), where *LIN28B* accelerates its own protein expression via inhibition of let-7 miRNAs [18]. LIN28B signals through repression of the let-7 miRNAs and consequently results in elevated MYCN protein expression in NB cells (Figure 4).

This complex signaling, involving LIN28B, let-7 and MYCN, blocks the differentiation of normal neuroblasts and NB cells, contributing to oncogenesis of NB. These findings were fully recapitulated in a transgenic mouse model in which *LIN28B* expression in the sympathetic adrenergic lineage induced the development of NB marked by low let-7 miRNA levels and high MYCN protein expression [18].

However, LIN28B is dispensable in *MYCN*-amplified neuroblastoma cell lines, despite de-repression of let-7 [21]. This because *MYCN* mRNA levels in amplified NBs are very high and sufficient to sponge let-7 and MYCN mRNA represents a preferred target that, in abundance, can sequester and impair let-7. Thus, NB employs multiple mechanisms to neutralize let-7 either by high expression of *LIN28B*, *MYCN* sponging, or genetic loss, placing let-7 disruption at the center of NB development [21]. Notably, a substantial expression of *LIN28B* in NBs without *MYCN* amplification has been detected that might result from epigenetic changes such as altered methylation or histone modification or the deregulation of upstream processes. Thus, high expression of *LIN28B* results in an adverse prognostic factor independent of *MYCN* amplification [18].

In NB primary tumors, a strong positive correlation has been identified between *LIN28B* expression and RAN (RAS-related nuclear protein) and AURKA (aurora kinase A) oncogenic proteins through both let-7-dependent and let-7-independent mechanisms [19]. LIN28B directly induces the expression of RAN, which in turn induces higher levels of phosphorylated AURKA and kinase activation. Moreover, AURKA is a direct let-7 target and stabilizes MYCN at the protein level, revealing a complex signaling cascade connecting LIN28B, RAN, AURKA and MYCN [19] (Figure 4).

Moreover, it has recently been demonstrated that overexpression of either wild-type LIN28B or a LIN28B mutant is unable to inhibit let-7 processing and increases the penetrance of MYCN-induced NB, potentiating the invasion and migration of transformed sympathetic neuroblasts and driving distant metastases in vivo [259].

Very recently, genome-wide ChIP-seq and co-immunoprecipitation experiments have shown that LIN28B indirectly binds the active gene promoters in NB cells through protein–protein interactions with the sequence-specific zinc-finger transcription factor ZNF143 [259] and acts as a cofactor to upregulate expression of a subset of downstream target genes, including those encoding transcription factors that comprise the ADRN CRC, such as HAND2, ISL1, PHOX2B, GATA3, TBX2 and MYCN [51], along with other directly up-regulated genes that are essential for NB cell survival, migration of the NC-derived lineages, cancer cell migration and invasion [259]. These findings reveal an unexpected let-7-independent function of LIN28B in transcriptional regulation of the ADRN core regulatory circuitry that controls the malignant cell state in NB during neuroblastoma pathogenesis. Thus, LIN28B may also promote MYCN-induced NB by a mechanism that does not require suppression of let-7 but instead is due to LIN28B-mediated upregulated expression via interaction with ZNF143 [259] (Figure 5).

## 7. Discussion: Pathologic Dysregulation of miR-34a, let-7b, miR-204 and *MYCN*, *PHOX2B*, *ALK* and *LIN28B* in Neuroblastoma

Given the complex interplay between miRNAs and TFs, their deregulation in NB has dramatic direct and indirect consequences on their regulatory loops, their downstream TGs and all the functional implications. Mutations and overexpression, due to gene amplification transcriptional and post-transcriptional dysregulation, play a fundamental role in the complex network of interactions among miRNA, TFs and TGs in NB (Table 1).

During the physiologic regulation, miR-34a, let-7b and miR-204 trigger an effective inhibitory regulation over MYCN, PHOX2B and LIN28B (Figure 3).

miR-34a is transcriptionally activated by the TP53 family [107] and exerts its suppression activity on *SIRT1* [108] and *MYCN* [36,116] mRNAs (Figure 3). miR-34a/SIRT1/p53 forms a positive feedback loop, wherein p53 induces miR-34a and miR-34a activates p53 by inhibiting SIRT1 with the consequent increased levels of acetylated p53 that leads to the activation of p53 pro-apoptotic target proteins. The reduced levels of miR-34a in NB lead to augmented levels of SIRT1 that maintains de-acetylated p53 (Figure 4, TP53*) with anti-apoptotic and pro-survival effects.

Reduced levels of miR-34a also affect *MYCN* expression, leading to an increase in MYCN protein levels. The higher levels of SIRT1 also stabilizes MYCN protein by creating a positive feedback loop via repression of MKP3, phosphorylation of ERK protein, which in turn phosphorylates MYCN [110] (Figure 4). Moreover, activation of MYC TFs leads to a widespread repression of miRNA expression, including miR-34a and let-7 family members, in a feedback manner [196] (Figure 4). The global effect of miR-34a underexpression is a MYCN-driven enhanced proliferation.

Underexpression of the let-7 miRNA family, observed in many cancers, is effected by the RNA binding proteins LIN28A/B that block pre-let-7 (inactive) processing to mature let-7 and their TS function [129] (Figure 4).

TP53 directly associates with AGO2 to induce or reduce the loading of a subset of miRNAs, including let-7 members; therefore, their cellular abundance or differential association with AGO2 are involved in an intricate network of regulatory feedback and feedforward circuits [141], including the regulation of LIN28A/B levels via let-7 (Figure 3 and Figure 4).

As let-7b targets both *MYCN* [34] and *LIN28B*, which negatively controls let-7 biogenesis [137]. In NB, reduced levels of let7 activate a feed-forward loop where *LIN28B* accelerates its own protein expression via inhibition of let-7 [18,137] (Figure 4). LIN28B further signals through the repression of let-7 and this results in elevated MYCN protein expression in NB cells [18]. *MYCN* in amplified NBs is so highly expressed that *MYCN* mRNA sponges let-7 (Figure 4). Thus, let-7 disruption, either by high expression of *LIN28B*, *MYCN* sponging, or genetic loss, has broad implications for NB development [21].

Moreover, LIN28B induces the expression of RAN, which in turn induces higher levels of phosphorylated AURKA and kinase activation that stabilize MYCN at the protein level, generating a LIN28B, RAN, AURKA, and MYCN signal cascade [19] (Figure 4).

As already reported, *LIN28B* is also a transcriptional target of MYCN [20]. Therefore, when MYCN is amplified/overexpressed, LIN28B expression also increases and blocks let-7 biogenesis, with consequent let-7 dependent de-repression of MYCN, thus establishing a direct MYCN/LIN28B/let-7 regulatory axis and activated feedback loops that greatly contribute to NB tumorigenesis [18,19,20,21] (Figure 4).

Additionally, LIN28B activates MYCN transcription in NB by indirectly binding its promoter through a protein–protein interaction with the zinc-finger ZNF143 TF [259] (Figure 5).

miR-204 may play a double role in cancer, acting as TS or oncomiR depending on the tumor [149]. miR-204 has been found to directly target *PHOX2B* [23,38] and *MYCN* [39] (Figure 3). As miR-204 is under-expressed in NB, its TS functions over the two TFs that have a powerful oncogenic role in NB are lost (Figure 4). Moreover, a double negative feedback loop has also been demonstrated between miR-204 and MYCN; consequently, when *MYCN* is overexpressed, it represses miR-204 and self-perpetuates [39] (Figure 4). This MYCN-mediated repression of miR-204 transcription could explain the low miR-204 expression in high grade MYCN-amplified NB cases [39]. In addition, miR-204 targets SIRT1 [150,151], reinforcing the inhibitory function of miR-34a over the gene (Figure 3), that is lost when miR-204 is under-expressed (Figure 4), feeding a loop miR-204/SIRT1/p53 similarly to the action of miR-34a.

In physiologic regulation, no direct interaction between MYCN and PHOX2B is documented. Conversely, in MYCN-amplified NB a small number of essential TFs belonging to the ADRN CRC, including PHOX2B, has demonstrated dependencies on SE MYCN-mediated expression [51]. Therefore, when amplified/overexpressed, MYCN acts as an enhancer invader [7] that reinforces the gene expression program of the entire ADRN CRC, which also promotes the enhancer/SE-driven transcription of PHOX2B (Figure 4 and Figure 7B).

ALK is a transcriptional target of PHOX2B [222] and MYCN [16]; therefore, overexpressed PHOX2B or amplified/overexpressed MYCN leads to increased levels of ALK. In turn, deregulated ALK generates a positive feedback loop by inducing an ERK5-mediated MYCN transcription [242,243] (Figure 4) through the PI3K, AKT, MEK3 and MEK5 signaling pathways, thus potentiating the oncogenic activity of MYCN.

As has already been remarked, MYCN has hundreds of targets, either coding or non-coding genes, two examples of which are reported in Figure 4, the TF and oncogene TWIST1 and the oncogenic miR-17-92 cluster.

Noteworthy is TWIST1, a known transcriptional target of both MYCN and MYC [210] and a regulator of mesenchymal lineage and the dedifferentiated cell state in NB [41,42]. TWIST1 directly activates its TGs by promoter binding, including PRRX1 (Figure 4), but also specifies other TGs relying on other cues, including potentially MYCN, to enforce transcriptional activation or repression by enhancer co-occupancy, therefore, it can be considered a deregulated MYCN-specific dependency in NB [7]. The oncogenic co-operation of MYCN and TWIST1 as enhancers demarcates a set of developmental genes important to NB tumorigenesis and highly sensitive to both MYCN and TWIST1 perturbation.

## 8. Conclusions and Perspectives

Although we have focused only on a small window of interactions among a few miRNAs and key oncogenes in NB whose deregulation over each other and over the downstream TGs has evident dramatic effects. The effects of the disruption of physiological regulation over MYCN, which results in its overexpression with the consequent generation of aberrant transcription activations and feedback loops on the hundreds of TGs that MYCN controls, are particularly powerful.

The discovery of NB-specific CRCs and related TFs that drive oncogenic transformation and maintain the malignant state and the knowledge of complex and strictly regulated networks impaired in NB, highlighting targetable oncogenic vulnerabilities in NB cells and opening up new perspectives on the design of innovative therapies specifically addressed to the genetic and epigenetic determinants of NB.

miRNA replacement approaches aimed at restoring TS function in NB have been successfully employed in mouse models through the combined delivery of miR-34a and let-7b mimics by NB-targeted nanoparticles [124]. Nevertheless, given the tight connections and the fine regulation among the four strong oncogenes described here (*MYCN*, *PHOX2B*, *ALK* and *LIN28B*), a more powerful therapeutic targeting of these genes should be leveraged to interfere with/block their oncogenic functions.

The use of protein small inhibitors, even when effective, induces drug resistance (i.e., ALK inhibitors [260]) and requires rather high doses that may induce unwanted toxicities in young patients. Moreover, inhibitors show different efficacies towards wt or mutated protein products.

Thanks to a sequence-specific design, RNAi-mediated knockdown can selectively target either wt or the mutated gene transcripts. This results in an extremely effective prevention of translation into protein. Thus, for the silencing of *ALK* expression in pre-clinical models of NB, we have successfully used NB-targeted liposomes entrapping siRNA against *ALK*, either alone [15,254] or in combination with an ALK inhibitor [255].

MYCN is still considered ‘undruggable’ but given its central role as am orchestrator in networks herein described, and the strong consequences of its deregulation in NB that lead to tumorigenesis and maintenance of a very aggressive tumor phenotype, it remains the most coveted target.

Different approaches to targeting MYCN have recently been reviewed, including strategies aimed at the suppression of MYCN transcription, destabilization of MYCN protein, e.g., inhibition of molecules stabilizing MYCN or preventing its degradation, inhibition of MYCN transcriptional activity, repression of MYCN co-factors and downstream targets and utilization of MYCN overexpression-dependent synthetic lethality [261].

Attempts to directly and specifically target MYCN mostly failed due to its similarity to c-MYC, the unstructured nature of the MYC family proteins in their monomeric forms, the lack of an understanding of MYCN-interacting proteins, the inability to obtain structural information on MYCN protein complexes, and the challenges of using traditional small molecules to inhibit protein–protein or protein–DNA interactions [262].

As an alternative to protein targeting, the direct targeting of MYCN mRNA using antisense molecules (siRNAs or shRNAs) for the treatment of MYCN-driven tumors could be advantageous. In the case of genes highly expressed, shRNAs would be very effective by providing a continuous knockdown of the target mRNA. Nevertheless, for systemic administration, more efficient and safe delivery systems should be developed to avoid viral-based technologies.

Remodeling aberrant regulatory networks from dysregulated gene expression that blocks differentiation and enhances proliferation toward a controlled expression of the disease-related oncogenes may represent a promising ‘gene-therapy’ approach for NB.

Since there is strong evidence that TFs are involved in the resistance to cytotoxic drugs [263], the targeting of CRC TFs may be relevant to sensitize NB cells to chemotherapy and may be beneficial for high-risk NB patients with refractory disease or chemo-resistant relapses.

Combinatorial strategies that integrate RNAi-mediated approaches (miR-mimics/anti-miRs, siRNAs/shRNA) with conventional chemotherapy and immunotherapy may lead to clinical applications to obtain a more effective therapeutic response.

## Figures and Tables

**Table 1:** Summary of the molecular machanisms underlying TF/TG dysregulated expression in NB.

TF/TG	MUTATIONS	OVEREXPRESSION		
		Transcriptional Activation	Gene Amplification	miRNA Downregulation
MYCN	rare	X	X	miR-34, let-7, miR-204
ALK	X	X	X	miR-96
PHOX2B	X	X	nd	miR-204
LIN28	rare	X	rare	let-7

X: detected; nd: not detected.

## Abbreviations

CCHS	Congenital Central Hypoventilation Syndrome
ChIP-seq	Chromatin Immunoprecipitation Sequencing
E10.5, E11.5	Embryonic Developmental Mouse Timeline Expressed in Days
EMT	Epithelial-to-Mesenchymal Transition
ESCs	Embryonic Stem Cells
FS	Frameshift
HD	Homeodomain
HSCR	Hirschsprung Disease
iPSC/s	Induced Pluripotent Stem Cell/s
miRNA	microRNA
MRX34	Liposomal Formulation of miR-34a Mimics
MS	Missense
NC	Neural Crest
NCCs	Neural Crest Cells
NICDs	Notch Intracellular Domains
NSCs	Neural Stem Cells
RNAi	RNA Interference
scRNA-seq	Single-Cell RNA Sequencing
shRNA	Short Hairpin RNA
siRNA	Small Interference RNA
SNPs	Single Nucleotide Polymorphisms
SNS	Sympathetic Nervous System
UTR	Untraslated Region
AGO2	Argonaute RISC Catalytic Component 2
AKT	Serine/Threonine Kinase
ALK/ALK	Anaplastic Lymphoma Kinase (receptor tyrosine kinase)
ALKAL2	ALK Additionally, LTK Ligand 2
AP-1	Activator Protein-1
Ascl1/ASCL1	Achaete-Scute Family bHLH Transcription Factor 1
BANCR	BRAF-activated non-protein coding RNA
bHLH	Basic Helix-Loop-Helix
BLIMP1	B-Lymphocyte-Induced Maturation Protein 1
BMI1	BMI1 Polycomb Ring Finger Proto-Oncogene
BMP	Bone Morphogenetic Protein
CCL2	C-C Motif Chemokine Ligand 2
CCNA2	Cyclin A2
CCNG2	Cyclin G2
CDK4	Cyclin dependent kinase 7
CDKL5	Cyclin Dependent Kinase Like 5
CDKN1A	Cyclin Dependent Kinase Inhibitor 1A
CHK1	Checkpoint Kinase 1
c-Myc/c-Myc	c-MYC proto-oncogene; homolog of v-myc avian myelocytomatosis viral related oncogene (hematopoietic cancer-derived), bHLH Transcription Factor
CRMP1	Collapsin Response Mediator Protein 1
c-SRC	SRC Proto-Oncogene, Non-Receptor Tyrosine Kinase
CYP26A1	Cytochrome P450 Family 26 Subfamily A Member 1
DBH	Dopamine Beta-hydroxylase
DDC	DCC Netrin 1 Receptor
DKC1	Dyskerin Pseudouridine Synthase 1
DKK1	Dickkopf WNT Signaling Pathway Inhibitor 1
DLL3	Delta Like Canonical Notch Ligand 3
E2F8	E2F Transcription Factor 8
EFNB3	Ephrin-B3
EGFR	Epidermal Growth Factor Receptor
ERK or MAPK1	Mitogen-Activated Protein Kinase 1
ERK3 or MAPK12	Mitogen-Activated Protein Kinase 12
ERK5 or MAPK7	Mitogen-Activated Protein Kinase 7
FAK	Focal Adhesion Kinase
FGF	Fibroblast Growth Factor
FOS	Fos Proto-Oncogene
Foxd3	Forkhead Box D3
FZD7	Frizzled Class Receptor 7
GAP43	Growth Associated Protein 43
Gata2/GATA2	GATA Binding Protein 2
Gata3/GATA3	GATA Binding Protein 3
Hand2/HAND2	Heart and Neural Crest Derivatives Expressed 2
HDAC1	Histone Deacetylase 1
HES	Hairy and Enhancer of Split
HMGA2	High Mobility Group AT-Hook 2
HRAS	Harvey Rat Sarcoma Virus Proto-Oncogene
HuC	RNA-Binding Protein HuC
HuD	RNA-Binding Protein HuD
ID2	Inhibitor of DNA Binding 2
IL-6	Interleukin 6
INHBA	Inhibitor of DNA Binding 2
ISL1	Islet LIM Homeobox 1
JUN	Jun Proto-Oncogene
Klf2, KLF2	Kruppel Like Factor 2
Klf4, KLF4	Kruppel Like Factor 4
KRAS	Kirsten Rat Sarcoma Virus
Lif	LIF Interleukin 6 Family Cytokine
LIN28A	Lin-28 Homolog A
Lin28b/LIN28B	Lin-28 Homolog B
LMO1	LIM Domain Only 1
LMO4	LIM Domain Only 4
MAML3	Mastermind Like Transcriptional Coactivator 3
MAPK	Mitogen-Activated Protein Kinase
MAX	MYC Associated Factor X
MCM	Minichromosome Maintenance Protein Complex
MCP-1	Monocyte Chemoattractant Protein-1
MDM2	Murine Double Minute 2
MEIS	Meis Homeobox
MEK3 or MAPK3	Mitogen-Activated Protein Kinase 3
MEK5 or MAPK5	Mitogen-Activated Protein Kinase 5
MET	MET Proto-Oncogene, Receptor Tyrosine Kinase
Miz-1/MIZ-1	Zinc Finger MIZ-Type Containing 1
MKP3	Mitogen-Activated Protein Kinase Phosphatase 3
MSX1	Msh Homeobox 1
MYBL2	MYB Proto-Oncogene Like 2
MYC	MYC Transcription factor family, bHLH
Myc-ERT	Tamoxifen-Inducible Myc
MYCL	MYCL Proto-Oncogene; homolog of v-myc avian myelocytomatosis viral related oncogene (lung cancer-derived), bHLH Transcription Factor
Mycn/MYCN	MYCN Proto-Oncogene; homolog of v-myc avian myelocytomatosis viral related oncogene (neuroblastoma-derived), bHLH Transcription Factor
Nanog	Nanog Homeobox
NeuroD1	Neuronal Differentiation 1
NFKB	Nuclear Factor Kappa-B
Notch/NOTCH	Notch Receptor 1
NTRK2	Neurotrophic Receptor Tyrosine Kinase 2
Oct3/4	Octamer-Binding Transcription Factor 3/4
p21	alias Cyclin Dependent Kinase Inhibitor 1A (CDKN1A)
P75NTR	p75 Neurotrophin Receptor
PC12	Pheochromocytoma Cells 12
PD-L1	Programmed Cell Death 1 Ligand 1
Phox2a/PHOX2A	Paired Like Homeobox 2A
Phox2b/PHOX2B	Paired Like Homeobox 2B
PI3K	Phosphatidylinositol 3-Kinase
PRRX1	Paired Related Homeobox 1
PUMA	p53 Upregulated Modulator of Apoptosis
RAN	RAN, Member RAS Oncogene Family
RARB	Retinoic Acid Receptor Beta
RAS	Rat Sarcoma Oncogene
RET	Rearranged During Transfection
RUNX1	RUNX Family Transcription Factor 1
SKP2	S-Phase Kinase Associated Protein 2
SNAI1	Snail Family Transcriptional Repressor 1
Snail2/SNAI2	Snail Family Transcriptional Repressor 2
Sox2	SRY-Box Transcription Factor 2
Sox 9	SRY-Box Transcription Factor 9
Sox10/SOX10	SRY-Box Transcription Factor 10
SP1	Specificity Protein 1
SSEA-1	Stage-Specific Embryonic Antigen 1
TBX2	T-box Transcription Factor 2
TG2	Tissue Transglutaminase 2
TGF-β1 (TGFB1)	Transforming Growth Factor Beta 1
TH	Tyrosine Hydroxylase
TIMP2	Tissue Inhibitor of Metalloproteinases 2
TP53	Tumor Protein P53
TP53INP1	Tumor Protein P53 Inducible Nuclear Protein 1
Tra2β	Transformer 2 Beta Homolog
Trk	Tropomyosin Receptor Kinase
TrkB/TRKB	Tropomyosin-Related Kinase B
TWIST1	Twist Family bHLH Transcription Factor 1
VEGF	Vascular Endothelial Growth Factor
Wnt/WNT	Wingless-Type MMTV Integration Site Family
ZCCHC11	Zinc Finger CCHC Domain-Containing Protein 11
ZEB1	Zinc finger E-box Binding homeobox 1
ZNF143	Zinc Finger Protein 143
